# The Orthologue of Sjögren's Syndrome Nuclear Autoantigen 1 (SSNA1) in *Trypanosoma brucei* Is an Immunogenic Self-Assembling Molecule

**DOI:** 10.1371/journal.pone.0031842

**Published:** 2012-02-20

**Authors:** Helen P. Price, Michael R. Hodgkinson, Rachel S. Curwen, Lorna M. MacLean, James A. Brannigan, Mark Carrington, Barbara A. Smith, David A. Ashford, Meg Stark, Deborah F. Smith

**Affiliations:** 1 Centre for Immunology and Infection, Department of Biology, University of York, Heslington, York, United Kingdom; 2 York Structural Biology Laboratory, Department of Chemistry, University of York, Heslington, York, United Kingdom; 3 Department of Biochemistry, University of Cambridge, Tennis Court Road, Cambridge, United Kingdom; 4 Technology Facility, Department of Biology, University of York, Heslington, York, United Kingdom; University of Texas-Houston Medical School, United States of America

## Abstract

Primary Sjögren's Syndrome (PSS) is a highly prevalent autoimmune disease, typically manifesting as lymphocytic infiltration of the exocrine glands leading to chronically impaired lacrimal and salivary secretion. Sjögren's Syndrome nuclear autoantigen 1 (SSNA1 or NA14) is a major specific target for autoantibodies in PSS but the precise function and clinical relevance of this protein are largely unknown. Orthologues of the gene are absent from many of the commonly used model organisms but are present in *Chlamyodomonas reinhardtii* (in which it has been termed DIP13) and most protozoa. We report the functional characterisation of the orthologue of SSNA1 in the kinetoplastid parasite, *Trypanosoma brucei*. Both TbDIP13 and human SSNA1 are small coiled-coil proteins which are predicted to be remote homologues of the actin-binding protein tropomyosin. We use comparative proteomic methods to identify potential interacting partners of TbDIP13. We also show evidence that TbDIP13 is able to self-assemble into fibril-like structures both in vitro and in vivo, a property which may contribute to its immunogenicity. Endogenous TbDIP13 partially co-localises with acetylated α-tubulin in the insect procyclic stage of the parasite. However, deletion of the *DIP13* gene in cultured bloodstream and procyclic stages of *T. brucei* has little effect on parasite growth or morphology, indicating either a degree of functional redundancy or a function in an alternative stage of the parasite life cycle.

## Introduction

Primary Sjögren's Syndrome (PSS) is a highly prevalent autoimmune disease, affecting an estimated 0.5% of the population of the Western World. Key features of this chronic disorder are lymphocytic infiltration of the exocrine glands leading to impaired lacrimal and salivary secretion, and the production of autoantibodies. Extraglandular manifestations are common and the risk of Non-Hodgkin's lymphoma is 44-fold higher than in healthy individuals [1]. Despite its high prevalence, PSS has been neglected in terms of research and the multifaceted mechanisms leading to pathogenesis remain poorly understood.

The human protein Sjögren's Syndrome nuclear autoantigen 1 (SSNA1, also known as NA14) is a major specific target for autoantibodies in PSS [2], [3] but the precise function and clinical relevance of this protein are largely unknown. SSNA1 is a small protein (13 kDa) with a high coiled-coil content and has been found on primary cilia, basal bodies, centrosomes and at the plasma membrane [4], [5], [6], [7]. The protein has been identified as a binding partner of the microtubule-severing protein spastin, a member of the AAA ATPase family, which is encoded by the *SPG4* gene [8]. Mutations in the *SPG4* gene are implicated in approximately 40% of cases of the genetic disorder hereditary spastic paraplegia (HSP) [9]. SSNA1 has also been identified as a binding partner for the G-protein coupled receptor TPRA40, the expression of which is rapidly downregulated during hypoxia and reoxygenation in mammals [6].

Orthologues of SSNA1 are absent from the common eukaryotic model systems *Saccharomyces cerevisiae*, *Caenorhabditis elegans* and *Drosophila melanogaster* but are encoded by the genomes of the flagellated green alga *Chlamydomonas reinhardtii*, trematode worms (*Schistosoma spp*.) and protozoan parasites [4]. The *C. reinhardtii* orthologue of SSNA1 (known as Deflagellation Inducible Protein 13 or DIP13) was identified as the product of an upregulated transcript following mechanical deflagellation. DIP13 has a similar cellular distribution to SSNA1, localising to microtubules (MTs) in the flagellar axoneme, basal bodies and cytoplasm [4], [10]. Knockdown of the *DIP13* transcript by antisense expression results in the production of cells with multiple nuclei and flagella [4], while over-expression of a GFP-tagged form of DIP13 causes a defect in the assembly of full-length flagella, correlating with a marked decrease in the rate of flagellar regeneration following mechanical deflagellation [10]. Further, a recent study utilising siRNA high-content screening reported that knockdown of *SSNA1* in mammalian cell lines did not affect cilium assembly but significantly inhibited the ciliary localisation of signalling cargo molecules Gli3 and GFP-tagged SMO [7]. GFP-tagged SSNA1 was localised to the centrosome in all cell types analysed in the study and additionally, to the basal body in ciliated cells [7]. These data indicate that SSNA1/DIP13 may have more than one role in cell division and ciliary function.

In the current study, we report the characterisation of the SSNA1/DIP13 orthologue (which we have termed TbDIP13) in the protozoan parasite *Trypanosoma brucei*. The parasite protein is predicted to assume a largely coiled-coil configuration and demonstrates a striking ability to assemble into fibril-like structures under physiological conditions, both in vivo and in vitro. This intrinsic property may contribute to immunogenicity, both of the trypanosome protein in the bloodstream of the infected host and also of the human protein as an autoantigen. Further, an antibody response to TbDIP13 can be detected in human African trypanosomiasis (HAT) patients with circulating bloodstream form parasites, raising the possibility of a parasite-induced autoimmune response during infection. We report that TbDIP13 partially co-localises with acetylated α-tubulin in trypanosomes and we identify a number of potential interacting partners by the use of comparative proteomics (iTRAQ). Deletion of both alleles of the gene by homologous recombination in bloodstream and procyclic form parasites has little effect on cell growth or morphology, suggesting either a degree of functional redundancy or a function in an alternative stage of the parasite life cycle.

## Results

### Identification of a *T. brucei* DIP13 orthologue

As part of an ongoing study on *N*-myristoylated proteins in kinetoplastids, we identified a protein sequence encoded by the *T. brucei brucei* genome (Tb10.61.2720 from GeneDB Version 2.1), of which the C-terminus shared 33% identity with the *Chlamydomonas reinhardtii* flagellar protein DIP13 [11]. Regions of this sequence were also detected in studies to identify components of the *T. brucei* flagellar proteome [12], [13]. The annotated 22 kDa protein had a predicted *N*-myristoylation site and an extended N-terminus compared to other orthologues but further analysis revealed that the wrong start codon had been assigned to this gene in the *T. b. brucei* genome sequence (described in detail in Figure S1). The corrected ORF encodes a protein of 13.2 kDa and pI 8.0 with no predicted N-terminal modifications but extensive coiled-coil regions covering over 70% of the sequence (Figure S1C).

### Overexpression of TbDIP13 in BSF parasites

**Figure 1:**
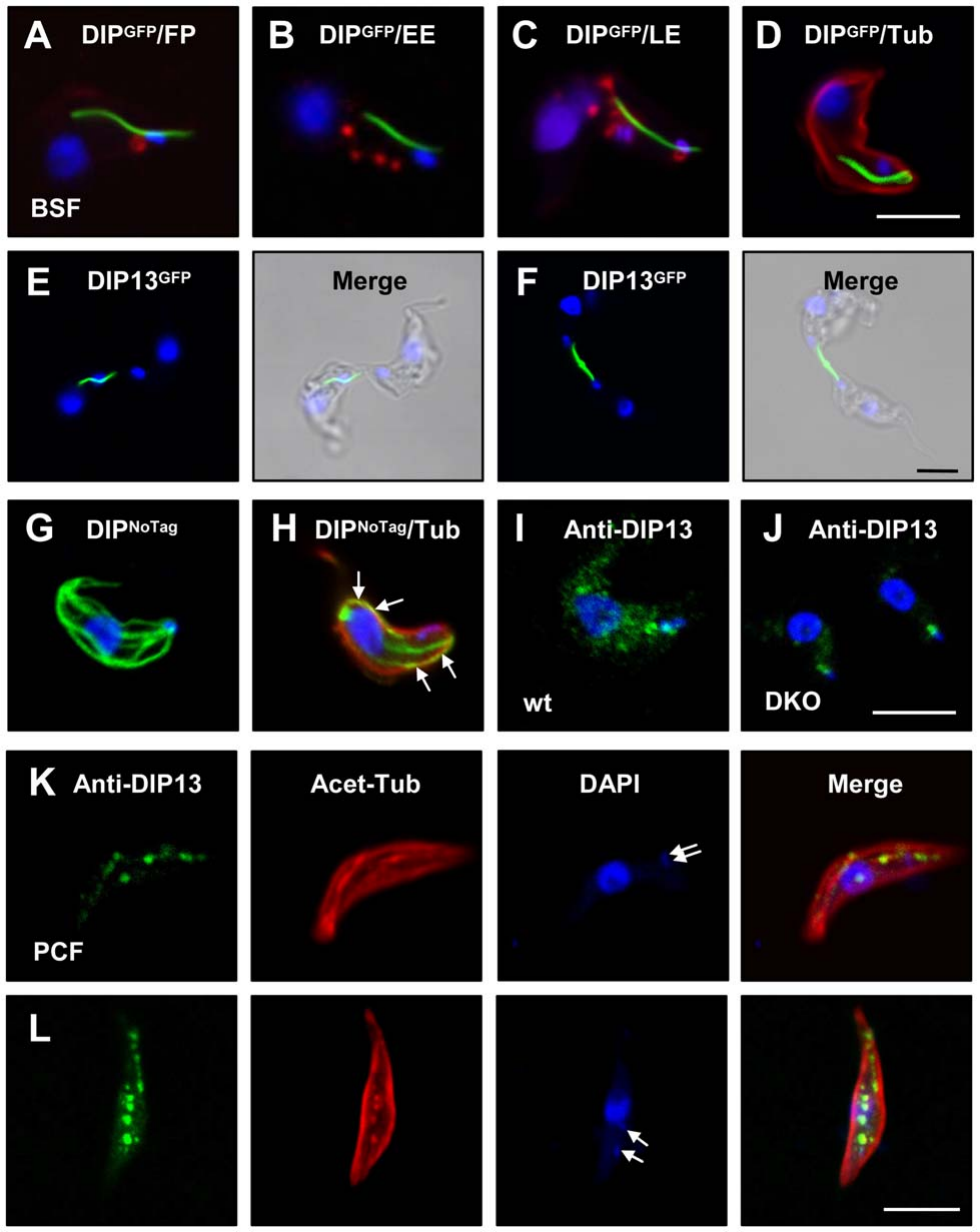
Subcellular localisation of DIP13 in *T. brucei*. (A–D) Fluorescence analysis of *T. brucei* bloodstream form (BSF) transgenic line 427/ pTbDIP13GFP grown in the presence of tetracycline for 24 hours. (A, C) Cells were prestained with Texas Red conjugated ConA (red) at 4°C to stain the flagellar pocket, FP (A) or at 37°C to stain the FP and endosomes (C). (B, D) Cells were probed with antibodies against the early endosome (EE) marker Rab5 (B) or α-tubulin (D). (E, F) Fluorescence analysis of *T. brucei* bloodstream form (BSF) transgenic line 427/pTbDIP13GFP (24 hours post-induction) undergoing cytokinesis, with corresponding DIC images. (G–J) Immunofluorescence analysis of transgenic line 427/ pTbDIP13^NoTag^ grown in the presence of tetracycline for 24 hours (G, H), BSF parental line Lister 427 (I) and *DIP13* double knockout line (J), all probed with purified rabbit anti-TbDIP13 (green). The cell shown in (H) was also probed with anti-α-tubulin (red) and regions of co-localisation are indicated by arrows. (K, L) Immunofluorescence analysis of *T. brucei* procyclic form (PCF) parental line EATRO 1125 probed with purified rabbit anti-TbDIP13 (green) and anti-acetylated α-tubulin (red) All cells were co-stained with DAPI (blue). Kinetoplasts are indicated by arrows. Bar, 5 µm.

In order to investigate the subcellular localization of TbDIP13, C-terminal epitope-tagged forms of TbDIP (TbDIP13^GFP^ and TbDIP13^myc^) were overexpressed using a tetracycline-inducible system in *T. brucei* bloodstream form (BSF) parasites (Figure 1A–D). It should be noted that the overexpression constructs were designed prior to splice-site mapping (see above) and contained a vector-derived trans-splice site, the accurate *TbDIP13* ORF with a C-terminal tag and an additional upstream region including the originally annotated start codon. Therefore there was a risk that an anomalous protein would be expressed. However, splice-site mapping following tetracycline induction of expression revealed that the parasites exclusively used the same splice site for the over-expressed transcript as for the endogenous transcript, thereby generating the template for the correct protein (Figure S1).

TbDIP13^GFP^ in BSF cells consistently localized to a long thin internal structure stretching from the far posterior end of the cell to a region close to the late endosomes (Figure 1C, D). There was no obvious association with the early endosomes (Figure 1B), paraflagellar rod, acidocalcisomes or glycosomes (data not shown). The mean length of the structure was 4.97 µm (+/− s.d. 1.36, n = 100) with an absolute range of 2.81–10.87 µm. The myc-tagged form of TbDIP (TbDIP13^myc^) had an identical localisation pattern (Figure S1D) showing that this unusual localisation was not caused by GFP aggregation. Further, full-length structures containing TbDIP13^GFP^ were visible in cells from 1 hour post-induction of expression and could be visualised by video microscopy in live cells (data not shown). Surprisingly, localisation of the protein appeared to be asymmetrical in a majority of cells (∼90%) in the late stages of cytokinesis, with a single filament evident in only one of the two daughter cells (Figure 1E). In a minor population of cells undergoing cytokinesis, the TbDIP13 structure was found at the site of abscission (Figure 1F), although the significance of this is not clear.

The majority of TbDIP13^GFP^ protein was highly insoluble and resistant to detergent extraction, remaining associated with the cytoskeleton following treatment with 1% NP40 and 1 M salt (Figure S2 and data not shown). Detergent-extracted cytoskeletons from cells over-expressing TbDIP13^GFP^ were probed with an anti-GFP antibody and observed by scanning electron microscopy to analyse the localization and origins of the novel structure at a higher resolution (Figure S2). TbDIP13 was uniformly distributed throughout the structure, which closely associated with the parasite cytoskeleton and appeared to be linked to the base of the flagellum. There was no significant effect on overall morphology or cell growth in TbDIP13^GFP^ expressing cells grown in the presence of tetracycline over a 5-day time course (Figure S3 and data not shown). Expression of TbDIP13^GFP^ was also attempted in PCF cells but no exogenous protein could be detected (data not shown).

### Localisation Studies using TbDIP13 Antibody

Polyclonal antibodies against the *C. reinhardtii* and human orthologues of SSNA1/DIP13 [2], [4] recognised multiple bands on immunoblots of *T. brucei* parasite lysates (data not shown) and so were unsuitable for TbDIP13 subcellular localisation studies. Therefore, we raised a polyclonal antibody against full-length recombinant TbDIP13 protein expressed in *E. coli*. Affinity-purified antibody was first used to analyse the subcellular location of overexpressed TbDIP13 without an epitope tag (TbDIP13^NoTag^). In contrast to tagged protein, TbDIP13^NoTag^ was detected in a number of long structures spanning the cell body (Figure 1G, H) which partially co-localised with α-tubulin (Figure 1H) consistent with an association with the cytoskeleton. However, endogenous TbDIP13 was detected in the BSF parental cell line in a diffuse punctate pattern, together with dense staining of a small region adjacent to the kinetoplast (Figure 1I). Staining of the diffuse punctate pattern was lost in *TbDIP13* null cell lines whereas the small intense region was retained (Figure 1J).

Our data for BSF cells therefore suggest that overexpressed TbDIP13 with the C-terminus blocked by an epitope tag oligomerises in a highly consistent manner to produce a single stable extraneous structure within the cell. In the absence of an epitope tag, TbDIP13 is seen on several long structures, indicating either the production of multiple extraneous structures such as that described above or binding of the protein to existing components of the cytoskeleton. In contrast, the endogenous protein is detected in a punctate pattern, suggesting either that oligomerisation does not occur at the normal intracellular concentration of the protein or that the observed puncta represent nucleating centres from which fine filaments radiate, which are undetectable by indirect immunofluorescence.

In contrast to our observations in BSF trypanosomes, endogenous TbDIP13 was found only in a subset of *T. brucei* procyclic form (PCF) cells with one nucleus and two kinetoplasts (i.e. in the early stages of mitosis). The protein was detected in a punctate pattern following one or more discrete lines which partially co-localised with acetylated α-tubulin (Figure 1K, L), as previously observed for *C. reinhardtii* DIP13 [4]. No equivalent staining with anti-TbDIP13 was seen in BSF parasites, suggesting a differential localisation of the protein in these two life cycle stages.

### SSNA1/DIP13 are remote homologues of tropomyosin

**Figure 2:**
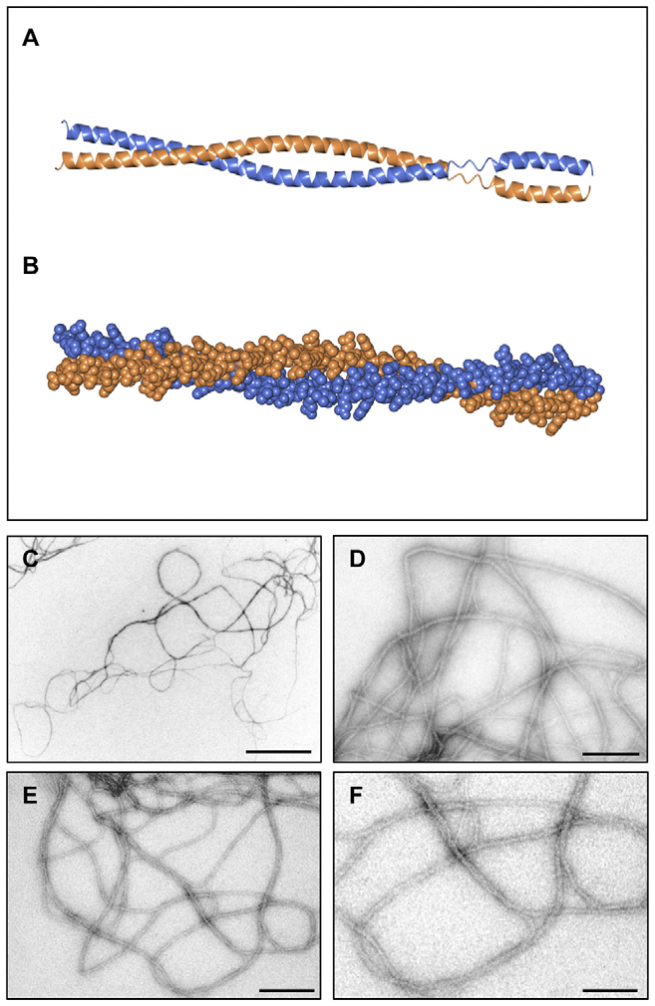
TbDIP13 protein analysis. (A–B) Molecular model of a homodimer of *T. brucei* DIP13, represented by ribbon (A) and sphere (B) drawings. Protein structural predictions were performed using the Phyre server and resulting data processed with CCP4mg and Coot software. The protein is predicted to dimerise to form a classical 2-strand parallel coiled-coil. (C–F) Transmission electron micrographs of recombinant TbDIP13 protein, following incubation in neutral pH buffer at 37°C overnight and staining with uranyl acetate. Bar, 500 nm (C), 200 nm (D, E) or 100 nm (F).

The protein structures of TbDIP13 and its orthologues in humans, *Chlamydomonas* and the protozoan *Leishmania major* were predicted using the Phyre server [14], [15] which combines a number of prediction programs and methods to identify remote homologues, predict secondary structure and build 3D models of a query sequence. All four of the SSNA1/DIP13 orthologues were predicted with high precision values (*e.g.* E-value of 3.5e-09 for human SSNA1) to be remote homologues of the actin-binding protein tropomyosin. Tropomyosin exists as a dimer of two α-helices arranged in a parallel coiled-coil which polymerise in a head-to-tail arrangement to form a long flexible rope-like filament [16]. The SSNA1/DIP13 orthologues were predicted to consist largely of α-helical coiled-coil with a short disordered region (∼20 amino acids) at the C-terminus. A 3D model of TbDIP13 (Figure 2A, B) predicts that the protein assumes a highly regular rod-like structure composed of a tightly wound parallel 2-strand coiled-coil, closely matching the known structure of tropomyosin despite low sequence identity between the two (14.8% at the amino acid level with human tropomyosin). These data therefore support our hypothesis that TbDIP may exist as a stable homodimer with the ability to oligomerise into filaments.

In order to produce direct evidence of homo-oligomerisation, purified recombinant TbDIP^His^ was incubated in a neutral pH buffer at 37°C overnight before analysis by negative stain transmission electron microscopy. The protein was clearly shown to self-assemble into filament-like structures reminiscent of amyloid fibrils (Figure 2C–F). The majority of these structures were approximately 10–15 nm wide and several thousand nm long. In addition to single filaments, coiled pairs of filaments were visible (Figure 2E) which may reflect the ability of the protein to bundle into larger structural components. The intrinsic properties of this oligomer-forming coiled-coil protein suggest that it may play a role in the assembly of filamentous structures.

### DIP13 is not essential for viability in *T. brucei*

**Figure 3:**
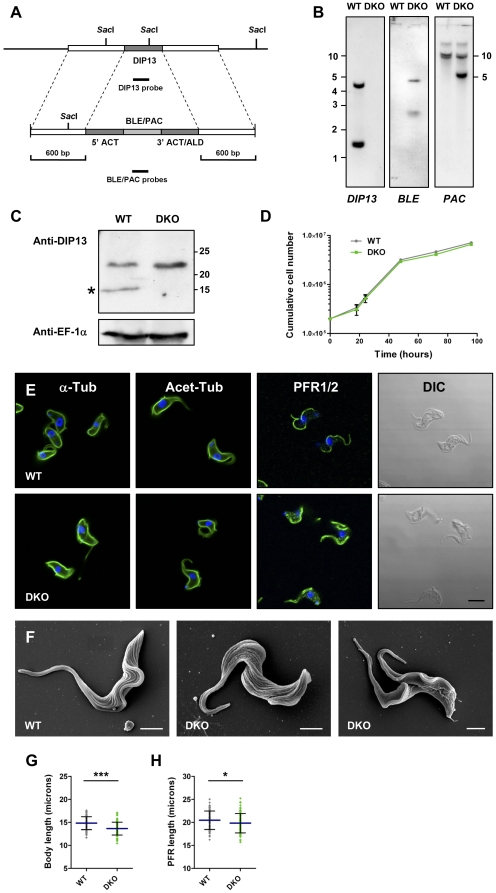
*DIP13* Gene Deletion in *T. brucei* BSF. (A) Schematic diagram of the *TbDIP13* locus and the plasmid constructs used for deletion. Flanking sequences used to generate the targeting vectors are shown. The top panel shows the *TbDIP13* locus and the second panel shows the plasmid constructs used for targeted deletion of the locus by replacement with phleomycin/puromycin resistance genes (*BLE*/*PAC*) flanked by the actin 5′ UTR and either the actin 3′ UTR (*BLE*) or the aldolase 3′ UTR (*PAC*). Solid black bars represent fragments used as hybridisation probes. (B) Southern blot analysis of parental line Lister 427 (WT) and *DIP13* double replacement (DKO) parasite lines. Five micrograms of genomic DNA from each parasite line was digested with *Sac*I, size separated through 0.8% agarose, blotted and hybridised with DIG-labelled DNA probes (∼200 bp) as indicated. Corresponding DNA marker positions are shown (kbp). (C) Total cell lysates (5×10^6^ cells/lane) from cell lines as in (B) were immunoblotted and probed with purified anti-TbDIP13 and anti-EF1α to monitor equal sample loading. Corresponding protein marker positions are shown (kDa). (D) Growth of BSF cell lines as above monitored over a 96 hour time course. Mean values are shown (n = 3) +/− SD (not all error bars are visible). (E) Immunofluorescence analysis of cell lines as above, probed with mouse monoclonal antibodies against total α-tubulin, TAT1 (α-Tub), acetylated α-tubulin, 6-11B-1 (Acet-Tub) and paraflagellar rod proteins 1 and 2, L13D6 (PFR1/2), shown in green. The corresponding DIC image is presented for cells probed with anti-PFR1/2. All cells were co-stained with DAPI (blue). Bar, 5 µm. (F) Scanning electron micrographs of parental (WT) and *DIP13* double replacement cell line (DKO). Bar, 2 µm. (G, H) Cell body length (G) and paraflagellar rod length (H) in BSF parental line Lister 427 (WT) and *DIP13* double replacement (DKO) parasite lines. Mean values are shown as horizontal lines (+/− SD) (n = 100). Statistical analyses were performed using an unpaired t test. Asterisks represent statistical significance compared to the parental line (WT) (* = p<0.05, *** = p<0.001).

**Figure 4:**
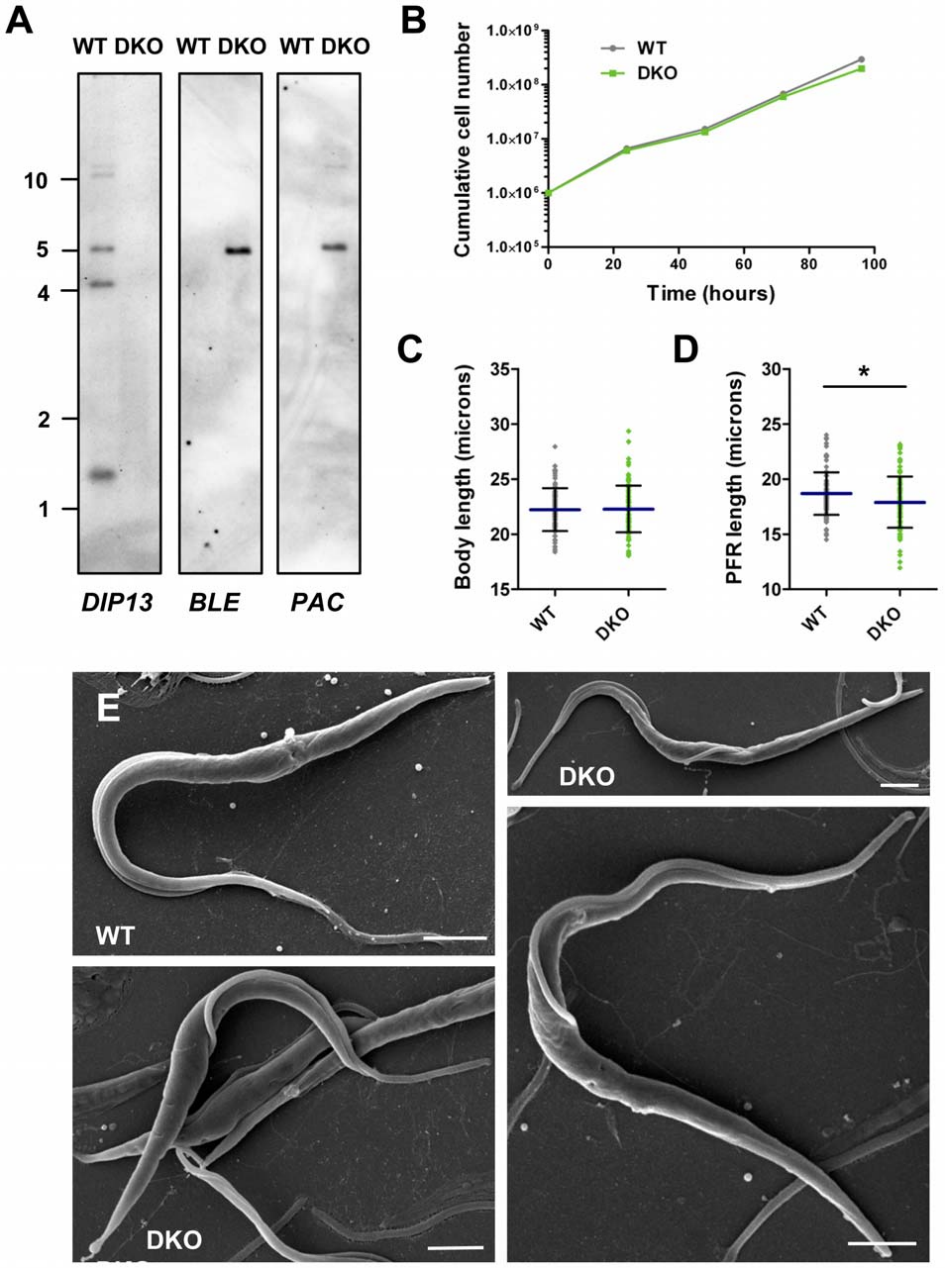
*DIP13* Gene Deletion in *T. brucei* PCF. (A) Southern blot analysis of parental line EATRO 1125 (WT) and *DIP13* double replacement (DKO) parasite lines. Five micrograms of genomic DNA from each parasite line was digested with *Sac*I, size separated through 0.8% agarose, blotted and hybridised with DIG-labelled DNA probes (∼200 bp) as indicated. Corresponding DNA marker positions are shown (kbp). (B) Growth of PCF cell lines as above monitored over a 96 hour time course. Mean values are shown (n = 3) +/− SD (not all error bars are visible). (C, D) Cell body length (C) and paraflagellar rod length (D) in PCF parental line EATRO 1125 (WT) and *DIP13* double replacement (DKO) parasite lines. Mean values are shown as horizontal lines (+/− SD) (n = 100). Statistical analyses were performed using an unpaired t test. Asterisks represent statistical significance compared to the parental line (WT) (* = p<0.05). (E) Scanning electron micrographs of parental (WT) and *DIP13* double replacement cell line (DKO). Bar, 2 µm.

RNA interference of *DIP13* was performed in *T. brucei* BSF but only partial knockdown (∼50%) was achieved by 24 hours and no phenotype was observed (data not shown). As an alternative approach, BSF and PCF null mutant strains were produced by homologous recombination (Figures 3 and 4), replacing both alleles of the *DIP13* gene with antibiotic resistance markers, resulting in the genotype Δ*dip13*::*BLE*/Δ*dip13*::*PAC* (Figures 3A, 3B and 4A). Double knockout lines were cloned by limiting dilution and tested for traces of *DIP13* DNA by the highly sensitive method of quantitative PCR (data not shown). Southern blots to confirm correct integration of the antibiotic resistance genes and presence/absence of *DIP13* are shown in Figures 3B and 4A. The *DIP13* probe hybridises to fragments of 4.4 and 1.2 kb in the BSF and PCF parental lines, with an additional band of approximately 5.5 kb in PCF which may correspond to partially digested DNA (Figure 4A). These bands are absent in the double knockout lines as expected. The *BLE* probe hybridises to a band of 5 kb in the double knockout cells and the *PAC* gene is found on a fragment of 4.8 kb in this line. Neither of these antibiotic resistance genes are detected in the parental line as expected but the *PAC* probe hybridises to additional cross-reacting fragments of 11 and 10 kb in both parental and knockout BSF lines, which may be due to non-specific cross-hybridisation with another region of the genome (Figure 3A).

Immunoblotting using anti-TbDIP13 demonstrates the loss of a 13 kDa band in the TbDIP13 BSF null line, with anti-EF-1α used as a constitutive loading control (Figure 3C). The anti-TbDIP13 antibody cross-reacts with an additional 22 kDa protein in both parental and null lines, which may represent the same molecule detected in a small intense region of the TbDIP13 null cells by immunofluorescence (Figure 1J).

No protein bands were detected in PCF cell lysates using anti-TbDIP13 (data not shown), which correlates with the small subset of cells found to express the protein by immunofluorescence analysis. As expected, no staining was detected when PCF cells from the knockout line were probed with the anti-TbDIP13 antibody (data not shown).

Deletion of both alleles of the *DIP13* gene had no significant effect on cell growth, as monitored over a 5-day period in BSF and PCF stage *T. brucei* (Figures 3D and 4B). Parasites showed normal motility (data not shown) and had no gross morphological defects as visualised by confocal and scanning electron microscopy (Figures 3E, F and 4E). Cells were stained with specific antibodies against total and acetylated α-tubulin, as a previous report suggested that *C. reinhardtii DIP13* may be preferentially associated with acetylated microtubules [4]. No differences were observed between parental and *TbDIP13* null lines in tubulin staining patterns and gross cell morphology (Figure 3E and data not shown). An antibody recognising paraflagellar rod proteins 1 and 2 produced more extensive staining in the *TbDIP13* null line (Figure 3E), with the emergence of a punctate pattern in addition to the characteristic PFR staining pattern. PFR1 and 2 are coiled-coil proteins and it is conceivable that in the null mutant line they are able to bind with low-specificity to structures or complexes instead of the absent TbDIP13 protein. However, no detrimental effects of this are seen and no structural changes to the flagellum can be seen in BSF and PCF stage parasites by scanning electron microscopy (Figures 3F and 4E) or transmission electron microscopy (data not shown). Parental and knockout parasites were stained with antibodies recognising α-tubulin or the paraflagellar rod proteins PFR1/2 and acquired images were used to measure cell body and PFR lengths in non-dividing cells (which have one nucleus and one kinetoplast). The PFR was found to extend to the distal tip of the flagellum in all cell lines (data not shown) and therefore could be used as a reliable marker of flagellum length. There was no significant difference in cell length in TbDIP13 knockout PCF parasites compared to the parental line (Figure 4C). There was however, a modest but statistically significant reduction in body length in the BSF TbDIP13 knockout line (mean value of 13.64 µm compared to 14.83 µm for the parental line, p = <0.0001) (Figure 3G). In addition, PFR length was significantly decreased in the TbDIP13 knockout lines of both life cycle stages compared to the appropriate parental line (p = <0.05) (Figure 3H and 4D). No defects in flagellum motility were observed (data not shown). We therefore conclude that under standard in vitro growth conditions, TbDIP13 is not essential in BSF and PCF cells for normal growth and morphology except for potential minor roles in the regulation of cell body and flagellum lengths.

We also tested the ability of the *DIP13* null BSF parasite line to establish an infection in a mouse model (data not shown). Mice were infected with 2×10^5^ parasites and levels of parasitaemia checked at 48 and 72 hours post-infection. By 72 hours, parasite concentrations in the blood were greater than 5×10^7^/ml in mice infected with either the parental or *TbDIP13* null line, indicating that TbDIP13 is not required for the successful establishment of a murine host infection.

### Immune recognition of *T. brucei* DIP13 in HAT Patients

**Figure 5:**
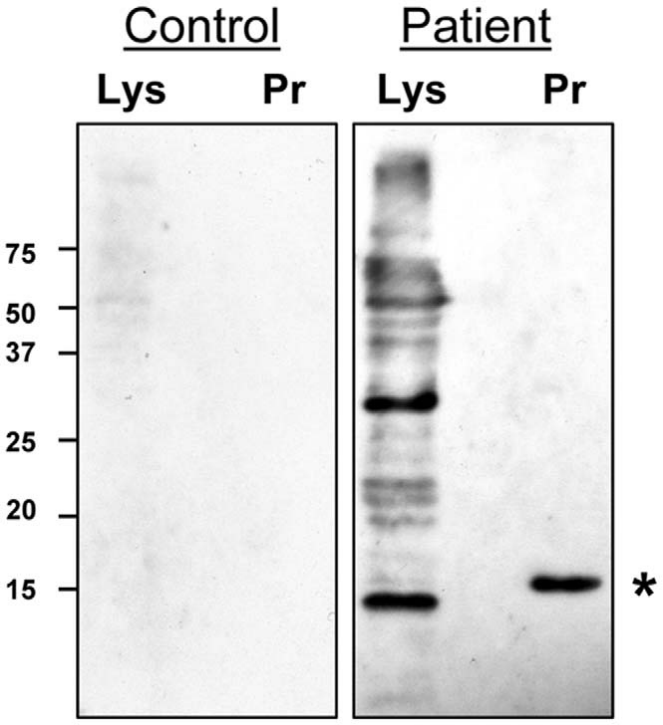
Immune Recognition of TbDIP13 by sera from human African trypanosomiasis (HAT) patients. Immunoblots were prepared using *T. brucei* BSF Lister 427 total cell lysate, Lys (5×10^5^ cells/lane) and recombinant TbDIP protein, Pr (30 ng/lane). Blots were probed with serum samples from HAT patients and healthy volunteers, with one example shown for each. The position of TbDIP13 protein is indicated by an asterisk. Corresponding protein marker positions are shown (kDa).

As the human orthologue of DIP13 is a known autoantigen, we looked for evidence that TbDIP13 is a dominant antigen in human African trypanosomiasis (HAT) patients, recognition that could potentially lead to an autoimmune response. Immunoblots of total BSF stage parasite lysate and purified recombinant TbDIP13 were probed with sera from 8 confirmed cases of *T. b. rhodesiense* (including both early and late stage disease) and 2 control samples from Uganda. Response patterns were highly variable and no single dominant antigen was strongly recognised by all patients. Antibodies to purified recombinant His-tagged TbDIP13 protein were detected with variable strength in 7 out of 8 patients but not in the control samples (Figure 5). The strongest response to recombinant TbDIP13 was detected in the serum of a HAT patient from the Tororo focus in SE Uganda, with early stage disease and a very high blood parasitaemia (wet film parasitaemia of 20 per 10 fields, ×400 magnification). This patient also responded strongly to a protein of the correct size (approximately 1 kDa smaller than the His-tagged recombinant protein) in total parasite lysate (Figure 5). Therefore TbDIP13 appears to be immunogenic, inducing the production of antibodies in HAT patients with detectable blood parasitaemia. However, the only patient sample displaying no anti-TbDIP13 response was from a late stage case of the disease with negative blood and positive CSF parasitaemia, implying that the antibody response against TbDIP13 is transient and characteristic of early infection. Further studies using a large representative panel of patient sera would be required to confirm these observations.

### Comparative proteomic (iTRAQ) analysis

Interaction studies can be problematic when working with highly insoluble proteins but here we exploited the ability of TbDIP13 to form stable insoluble structures, using our *T. brucei* BSF lines expressing DIP13^GFP^ or DIP13^myc^ as a model system to identify potential interacting partners of the protein. Human SSNA1 has been reported to bind to spastin [8] and TPRA40 [6] but no other binding partners of this protein are known. As described above, our data indicate that overexpressed TbDIP13 self-assembles into long structures which can be extracted with the highly insoluble flagellum/basal body components of the cell. We employed the proteomic non-gel technique isobaric tag for relative and absolute quantitation (iTRAQ), to identify and compare the relative amounts of the components of this insoluble fraction in TbDIP13 overexpressing cells compared to the parental line.

Flagellar extracts were prepared by consecutive detergent and high salt extraction steps, then redissolved in buffer containing 5 M urea and 0.5% SDS. Protein concentration was measured and confirmed by SDS-PAGE (Figure S4A). Following iTRAQ labelling and mass spectrometry, a total of 257 proteins were identified with at least 1 peptide with an ion score with higher than 95% confidence, representing the first published flagellar proteome of *T. brucei* BSF cells. No novel proteins were found in the TbDIP13 overexpressing cell extracts compared to the parental line. Full lists of identified proteins are provided in Tables S2 and S3. The data set was filtered to exclude proteins with a pI of 10.2 or greater as these are likely to be ribosomal contaminants (Table S3). The remaining data set contains 184 proteins, of which 121 have previously been found in the *T. brucei* PCF flagellar proteome [12], [13] and 63 are novel (Table S2). Included in the novel set of proteins are a number of expected flagellar components, such as a kinesin motor subunit (Tb10.61.1750) and dynein light chain (Tb11.03.0815), in addition to many hypothetical proteins. A major difference between BSF and PCF parasites is in metabolic requirements, with BSF entirely dependent on glycolysis for ATP [17] while PCF can utilise lipids and amino acids, particularly proline [18]. Several glycolytic enzymes were detected in the BSF flagellar extracts, possibly representing contamination from glycosomes, the organelles in which these enzymes are compartmentalised. In addition, several chaperone proteins were found in the BSF flagellum extracts, including BiP, DNAJ, HSP60 and HSP70. While these may also represent contaminants, previous reports have described subpopulations of abundant chaperones (including HSP70A and DNAJ) to localise specifically to flagella in *Chlamydomonas* and mouse spermatozoa, to associate with the intraflagellar (IFT) machinery and to have roles in flagellum assembly and function [19], [20], [21].

For quantitative analysis of our proteomic data, the number of peptide events was first plotted against iTRAQ ratios for two independent experimental replicates (cells expressing TbDIP13^myc^) and for experimental samples vs the parental control (Figure S4B, C). Based on data obtained for the experimental replicates, peptides detected in less than 4 events were removed from the iTRAQ data set. Of the remaining 106 proteins, 45 (in addition to TbDIP13) were found to be significantly enriched or reduced (P value<0.05) in at least one of the experimental samples compared to the parental extract, of which 17 were predicted to contain coiled-coil domains (Table S4). However, with the exception of TbDIP13 itself, no proteins were enriched more than 2-fold and only four (plus TbDIP13) were significantly enriched in all three TbDIP13 overexpressing samples compared to the parental control (Table 1). All four of these are included in the published *T. brucei* PCF flagellar proteome [12], [13] and two are kinetoplastid-specific hypothetical proteins with no known domains or motifs. Our data show that α-tubulin was significantly enriched and β-tubulin significantly reduced (both with extremely low P values) in the flagellar extracts from overexpressing lines compared to control, although the biological relevance of these observations is unclear. The fourth enriched protein is the kinetoplastid-specific microtubule-associated protein 1 (MARP1), which consists of more than 50 tandemly arranged 38 kDa repeat units (microtubule-binding motifs) flanked by short nonrepetitive N- and C-termini [22]. MARP1 is reported to be one of the major structural components of the *T. brucei* cytoskeleton and, like TbDIP13, is antigenic in the mammalian host [23], [24]. MARP1 could provide the necessary linkage between oligomerised TbDIP13 and cellular microtubules, unless TbDIP13 is able to bind directly to tubulin.

**Table 1:** Proteins identified by comparative proteomics (iTRAQ) as enriched in the flagellar extracts of *T. brucei* BSF cells overexpressing DIP13.

ACC. NO.	NAME	MW	pI	FEATURES	GFP/WT	PVal	Myc1/WT	PVal	Myc2/WT	PVal
Tb10.61.2720	Hypothetical protein (DIP13)	13.2	8.0	Coiled coil	4.253	0.000	5.767	0.000	5.292	0.000
Tb11.50.0001	Hypothetical protein	30.1	9.4	Unknown	1.478	0.000	1.474	0.016	1.736	0.010
Tb10.6k15.1500	Hypothetical protein	45.0	9.2	Unknown	1.263	0.002	1.580	0.000	1.626	0.000
Tb927.1.2400	Alpha tubulin	49.7	4.7	Tubulin, coiled coil	1.196	0.000	1.242	0.000	1.231	0.000
Tb10.406.0560	Microtubule-associated protein	237.4	5.2	Repeats	1.167	0.000	1.420	0.000	1.437	0.000

WT, BSF parental line Lister 427. GFP, cells overexpressing C-terminal GFP tagged DIP13. Myc1 and 2, experimental replicates of cells overexpressing C-terminal myc-tagged DIP13.

In addition to the molecules described above, a number of proteins were identified as enriched or depleted in some but not all three of the TbDIP13 overexpressing parasite samples (Table S4). There is a greater likelihood that GFP-fusion proteins will have impaired folding and functionality compared to proteins bearing the much smaller *myc* epitope tag [25], so it is possible that some interacting partners are able to bind to myc-tagged TbDIP13 but not to the GFP-tagged isoform. Six proteins were identified as enriched in both TbDIP13^myc^ expressing samples but not in the TbDIP13^GFP^ extract. These include three coiled-coil containing hypothetical proteins (Tb927.4.1300, Tb11.02.0210 and Tb927.7.2650) and a hypothetical protein containing leucine rich repeats (Tb11.01.8770). The remaining two proteins are a member of the calpain family of peptidases (Tb927.8.8330) and a hypothetical protein containing a C2 calcium-lipid binding motif (Tb927.7.3550).

While giving potential clues to DIP13 interactions, these data need to be interpreted with caution and further studies will be required to determine whether any of the enriched proteins are true binding partners of TbDIP13.

## Discussion

*T. brucei* DIP13, as characterised in this study, is a small coiled-coil protein which partially co-localises with acetylated tubulin in the insect stage procyclic form of the trypanosome and is able to self-assemble into filaments similar to its distant homologue, tropomyosin. Serum samples from patients infected with *T. brucei rhodesiense* have detectable levels of anti-TbDIP13 antibodies. However, deletion of the gene encoding TbDIP13 has little effect on parasite growth or morphology in either procyclic or bloodstream form parasites, with the exception of a modest decrease in cell size and flagellum length. Correlating with these observations, bloodstream form *TbDIP13* null parasites retain the ability to infect mice.

Alpha-helical coiled-coil motifs are found in an estimated 10% of all protein sequences [26], [27] including structural proteins (*e.g.* collagen), SNARE complex subunits, cytoskeletal motor proteins, transcription factors and numerous other groups [28], [29], [30], [31]. The archetypal coiled-coil protein, tropomyosin, has a highly regular α-helical structure composed of heptad repeats (denoted a-b-c-d-e-f-g) in which the first and fourth residues are hydrophobic. Pairs of tropomyosin molecules wind round each other to produce coiled-coils which are able to polymerise head-to-tail to form stable flexible filaments that associate with actin in striated muscle [16]. Our analyses predict that the orthologues of DIP13/SSNA1 in humans, *Chlamydomonas* and protozoa are structurally very similar to tropomyosin and to each other, despite low sequence identity, and therefore might have the intrinsic ability to assemble into a filament-like structure when present at sufficient concentration. This latter prediction is validated here both in vitro by transmission electron microscopy of purified recombinant TbDIP13 protein and in vivo by visualisation of overexpressed protein. A single filament was produced in parasites over-expressing TbDIP13 with a C-terminal epitope tag (either GFP or myc), which appeared to nucleate from the posterior end of the cell where it was linked to the cytoskeleton. In comparison, cells overexpressing untagged TbDIP13 contained a number of DIP13 filaments which partially co-localised to tubulin, suggesting that the C-terminus may be important for microtubule binding or in regulating oligomerisation. Endogenous TbDIP13 was detected at much lower concentration in the parasite and did not produce visible filaments. Instead, the protein was detectable in a diffuse punctate pattern in bloodstream form parasites and as specific puncta co-localising with a subpopulation of acetylated α-tubulin in insect stage parasites undergoing mitosis. It should be noted that in most eukaryotes, acetylated tubulin is associated with stable and not dynamic microtubules [32], [33] but this modified form of tubulin has a widespread distribution in *T. brucei* in all microtubule arrays, including the mitotic spindle [34]. The TbDIP13 filaments observed both in vitro and in vivo here appear to be extraneous but may reveal physical features of relevance to the physiological properties of the native protein, which could act as a flexible linker or scaffold within the cell.

The lack of a distinct phenotype in cultured trypanosomes following the deletion of *TbDIP13* suggests a degree of functional redundancy, with potential compensation by other coiled-coil proteins with similar structure. However, these results are surprising given that the single-copy genes encoding orthologues of SSNA1/DIP13 are present only in the Chordata, protists and plants. It is possible that the null mutant parasites are defective in functions that are critical at other stages in the life cycle that are not easily studied in culture, *e.g.* during development in the tsetse fly vector, including parasite meiosis which is non-obligatory and occurs at very low frequency in *T. brucei*, most likely in the salivary glands of the vector [35], [36]. The *T. brucei* Lister 427 BSF strain used in this study has been extensively cultured and is monomorphic, having lost the ability to differentiate in the host from replicating long slender forms to non-dividing short stumpy forms which are taken up by the tsetse fly [37]. In order to study the functions of TbDIP13 either in this differentiation process or in stumpy form parasites, further molecular work will be required using a pleomorphic strain of *T. brucei*. In addition, in vitro culture cannot be used to reproduce parasite differentiation from the procyclic (PCF) form found in the tsetse midgut through a number of intermediate forms in the proventriculus to the final host-infective metacyclic form which resides in the salivary gland of the insect vector [38]. Assessing the impact of TbDIP13 expression in these life cycle stages and their production will require experimental infection of tsetse flies with the generated PCF null line. In spite of these limitations, we can conclude from our current data that DIP13 is not required to maintain standard growth rates and morphology of *T. brucei* BSF and PCF stages in culture, or to initiate infection of a mouse model with long slender forms of the parasite.

As an alternative strategy to elucidate the functions of TbDIP13, we used comparative proteomic technology to identify putative binding partners of the protein. A similar application of this technique has been employed on extracts from *T. brucei* lines following RNAi knockdown of the paraflagellar rod (PFR) proteins and other insoluble flagellar components [39]. We identified a number of potential interactions, although further validation of these has been hampered by the tendency of isolated TbDIP13 to aggregate (data not shown). Further, pulldown studies using this protein (from over-expressing parasites, *E. coli* or generated by in vitro translation) have been challenging due to the harsh buffers required to maintain the protein in solution. Previous research showed that human SSNA1 is able to bind to the G-protein coupled receptor TPRA40 [6] and the microtubule-severing protein spastin [8]. G-protein coupled receptors are absent from kinetoplastid genomes [40], while although a homologue of spastin has been characterised in *T. brucei*, this is absent from the related protozoan *Leishmania major* [41] which has a DIP13 orthologue. Spastin in *T. brucei* localises to the nucleus [41] and does not appear to have complementary functions to TbDIP13. Knockdown of spastin expression by RNAi has no effect on growth or morphology, while overexpression of tagged protein results in nuclear enlargement [41]. Another MT-severing protein in *T. brucei*, fidgetin, localises to the nucleus during most of the cell cycle but relocates to dots, suggested to be kinetochores, on the mitotic spindle during mitosis [41], in a pattern similar to that of TbDIP13 in PCF cells. RNAi knockdown of a third type of MT-severing proteins, katenin, in *T. brucei* causes a significant reduction in flagellum length in *T. brucei*, as seen for the *DIP13* knockout cell lines [41]. None of the MT-severing proteins were found in our proteomic analysis and further investigation is needed to determine if TbDIP13 is a component of kinetochores and if there is any functional link between DIP13 and MT-severing proteins in trypanosomes.

Human SSNA1 and its orthologues are predicted to assume a coiled-coil conformation, a property shared with several other autoantigens associated with rheumatic disease, including the golgin family of Golgi-localised proteins (which are linked to systemic lupus erythematosus (SLE) and Sjögren's syndrome [42]) and the centrosomal protein, pericentrin [43]. The SSNA1 protein was originally identified as an autoantigen from a single Sjögren's syndrome patient [2] and a recent study showed that 14% of PSS cases tested positive for autoantibodies against this protein, compared to 2% or less of patients with other rheumatoid diseases. Therefore, SSNA1 is believed to be a minor but specific autoantigen of PSS [3]. The implications of this particular response and correlation with clinical manifestation are currently unknown but previous studies have linked autoantibody response profile to clinical manifestation of rheumatic diseases and may be used in diagnosis and prognosis [44], [45]. SSNA1 and its orthologues are structurally very similar to α-tropomyosin, which itself is an autoantigen associated with a subset of patients with the multi-system inflammatory disease Behçet's syndrome [46] and a major allergen associated with responses to invertebrates including house dust mites, cockroaches and shellfish [47]. Our findings that TbDIP13 can form stable filaments resembling amyloid fibrils may have relevance to SSNA1 (and possibly tropomyosin) as an autoantigen. It is possible that minor populations of patients showing an SSNA1 autoantibody response may have upregulated expression of this protein to the level at which aggregation can occur. Toxic protein aggregation is a common factor of many neurodegenerative diseases, including Alzheimer's and Parkinson's diseases, and other conditions such as dialysis-related amyloidosis [48], [49]. There is a characteristic accumulation of amyloid deposits in these disorders but cytotoxicity is believed to be largely due to intermediate oligomeric species which can be readily taken up into cells [50]. There are also several potential effects of aggregate formation on immunogenicity [51], which may be relevant for SSNA1. Protein multimers of over 100 kDa are much more efficient at inducing an immune response than monomers of low molecular weight proteins. Insoluble aggregates are more resistant to degradation, have better engagement with antigen-presenting cells and result in a more persistent response than soluble species [51]. Therefore it is likely that an insoluble multimer of SSNA1 would be more immunogenic than a soluble monomer. However, the antibodies produced may not necessarily recognise the native monomer, instead showing some specificity for the higher order structure of the aggregate [51] and potentially cross-reacting with other similar structures. It is of note in this paper that serum samples from patients diagnosed with *T. brucei rhodesiense* infection contain antibodies that recognise TbDIP13. Further work is required to assess whether these antibodies cross react with human SSNA1, which may lead to autoimmune complications in HAT patients.

## Materials and Methods

### Bioinformatics

Parasite genome sequence data were obtained from GeneDB [52] and TriTrypDB [53] resources. DIP13 protein structural predictions were performed using the Phyre server [15] and resulting information processed with CCP4mg [54] and Coot [55] software.

### Parasite Culture

The *T. brucei* bloodstream form strain Lister 427 was maintained in vitro as described [56]. The procyclic strain EATRO 1125 was maintained in vitro at 26°C in SDM79 medium [57] containing 10% tetracycline-free fetal bovine serum (Autogen Bioclear).

### DNA Constructs

All primer sequences are provided in Table S1. The plasmid vector pT7-^MYC-C^ [58], [59] was a gift from David Horn and Sam Alsford (London School of Hygiene and Tropical Medicine, London, United Kingdom). This vector contains flanking regions for integration into the transcriptionally silent rDNA spacer regions of the *T. brucei* genome and can be used to overexpress the target gene with a C-terminal myc epitope tag under the control of a tetracycline-inducible T7 promoter. The complete open reading frame of the *T. brucei DIP13* orthologue (Tb10.61.2720, amino acids residues 1–195 as annotated GeneDB Version 2.1) was amplified from genomic DNA using primers DIP-F1 and DIP-R1 and cloned into the plasmid vector pcDNA3.1/CT-GFP TOPO (Invitrogen). C-terminal GFP-tagged TbDIP13 was then amplified from the resulting construct, using primers DIP-F2 and DIP-R2, and cloned into *Hind*III/*Xba*I/Klenow fragment treated vector pT7-^MYC-C^ to produce construct pTbDIP13^GFP^. The *TbDIP13* open reading frame (as above) was also amplified using primers DIP-F3 and DIP-R3, digested with *Hind*III/*Xba*I and ligated into digested pT7-^MYC-C^ vector to produce the construct pTbDIP13^myc^. In addition, the regions encoding TbDIP13 residues 66–195 and 77–195 were amplified from genomic DNA (using primers DIP-F4 and DIP-R3; or DIP-F5 and DIP-R3, respectively). Both fragments were digested with *Hind*III/*Xba*I and ligated into digested pT7-^MYC-C^ vector as above, to produce the constructs pTbDIP13-Int^myc^ and pTbDIP13-Short^myc^. A construct for overexpression of untagged TbDIP13 (pTbDIP13^NoTag^) was produced by the introduction of a stop codon upstream of the C-terminal myc epitope tag in pTbDIP-Short^myc^ using the GeneTailor Site-Directed Mutagenesis System (Invitrogen) and primers DIP-F6 and DIP-R6.

The RNA interference plasmid vector p2T7Ti was a gift from Doug LaCount (PULSe, Purdue University, West Lafayette, IN, USA). A region spanning residues 167–585 of the *T. brucei DIP13* open reading frame (as annotated in GeneDB Version 2.1) was amplified from genomic DNA using the primers DIP-F7 and DIP-R7, digested with *Xba*I and cloned into *Xba*I-digested p2T7Ti [59] to produce the construct p2T7DIP13.

For production of *TbDIP13* null BSF lines, the phleomycin resistance gene *BLE* flanked by *ACT* 5′ and 3′ untranslated regions was amplified using primers BLE-F and BLE-R from plasmid vector pLew82v4 (a gift from George Cross, Laboratory of Molecular Parasitology, Rockefeller University, New York, USA). The resulting 1607 bp fragment was cloned into plasmid vector pCR2.1-TOPO (Invitrogen) to produce the construct pBLE-TOPO. A 570 bp region of *T. brucei* genomic DNA beginning 170 bp downstream of the *DIP13* ORF (as annotated in GeneDB Version 2.1) was amplified using primers DIP-F8 and DIP-R8. The product was digested with *Xho*I*/Xba*I and cloned into digested pBLE-TOPO to produce plasmid pBLE-DIP3UTR. A 564 bp region beginning 771 bp upstream of the *T. brucei DIP13* ORF was then amplified using primers DIP-F9 and DIP-R9, digested with *Sac*I/*Eco*RV and cloned into plasmid pBLE-DIP3UTR to produce the knockout construct pTbDIPKO-BLE. For replacement of the second allele of *DIP13*, pTbDIPKO-BLE was digested with *Eco*RV/*Xho*I to excise the *BLE* cassette. This was replaced by a 1039 bp fragment encoding the puromycin resistance gene *PAC* flanked by 5′ *ACT* and 3′ *ALD* untranslated regions, amplified from the plasmid vector PHD1034 (a gift from Christine Clayton, ZMBH, Universitat Heidelberg, Germany) using primers PAC-F and PAC-R to produce the knockout construct pTbDIPKO-PAC.

For protein expression in *E. coli*, a fragment spanning residues 77–195 of the *TbDIP13* open reading frame (as annotated in GeneDB Version 2.1) was amplified from genomic DNA using primers DIP-F10 and DIP-R10 and cloned into the plasmid vector pET101/D-TOPO (Invitrogen) to produce the construct pET-DIP13^His^.

### Antibody Production

The construct pET-DIP13^His^ was introduced into *E.coli* BL21 Star (DE3) and expression of recombinant protein was achieved by induction with 1 mM IPTG for four hours at 30°C. For large-scale protein purification, cells from 5 L culture were resuspended in 100 ml lysis buffer (8 M urea, 300 mM NaCl, 20 mM sodium phosphate pH 7.4, 40 mM imidazole and 1× Complete protease inhibitor cocktail, Roche). Cells were lysed by three rounds of sonication then centrifuged at 50,000 *g* for 40 minutes at 15°C. Purification was performed on an AKTA100 (GE) fitted with a direct loading pump. The clarified lysate was loaded directly onto an equilibrated 1 ml HisTrap crude column (GE) at a flow rate of 1 ml/minute. Following a 10 column volume (CV) wash with buffer A (8 M urea, 300 mM NaCl, 20 mM sodium phosphate pH 7.4, 40 mM imidazole), bound proteins were eluted with buffer B (8 M urea, 300 mM NaCl, 20 mM sodium phosphate pH 7.4, 0.5 M imidazole) using a gradient of 0–100% B over 10 CV. Fractions of 1 ml were collected and analysed by SDS-PAGE. Peak fractions were pooled and TCA precipitated. The pellet was redissolved in 6 M urea. The protein yield was approximately 2 mg/L cells.

Polyclonal antibodies were produced from two rabbits (Eurogentech, 87 day Classic protocol). Antibodies were purified using a 1 ml NHS-activated HP column (GE) coupled to 5 mg of recombinant TbDIP13 protein. Following column equilibration with 10 ml binding buffer (20 mM sodium phosphate pH 7.0, 150 mM NaCl), 15 ml rabbit serum was loaded onto the column at 0.3 ml/minute. Unbound sample was removed with a 5 ml wash with binding buffer. Elution was then performed using elution buffer at low pH (0.1 M glycine pH 2.7, 0.5 M NaCl). Fractions of 0.5 ml were collected directly into tubes containing 50 µl 1 M Tris-HCl pH 9.0 and analysed by SDS-PAGE. Peak fractions were pooled and tested by immunoblotting (1∶200). Antibodies against *C. reinhardtii* DIP13 [4] (a gift from Wolfgang Mages, Universität Regensburg, Germany) and human NA14 [2] (a gift from Rosa Rios Sanchez, CABIMER-CSIC, Seville, Spain) were also tested on immunoblots containing *T. brucei* parasite lysates at a range of concentrations but were unsuitable for further studies on TbDIP13.

### DIP13 Overexpression in *T. brucei* BSF

The constructs pTbDIP13^GFP^, pTbDIP13^myc^, pTbDIP13-Int^myc^, pTbDIP13-Short^myc^ and pTbDIP13^NoTag^ were transfected into mid-log phase *T. brucei* BSF using the Nucleofector® system as described [60]. Expression of epitope-tagged or untagged DIP13 was induced in stable cell lines by incubating parasites in 1 µg/ml tetracycline for 0–96 hours. Immunoblotting of total parasite lysates was performed as described [59]. GFP expression was analysed by flow cytometry using a Dako CyAn with FL1 detector and results analysed with Summit v4.3 software.

### Splice site Mapping

TRIzol Reagent (Invitrogen) was used to extract total RNA from parasites (*T. b. brucei* Lister 427 parental line and cells stably transfected with pTbDIP-GFP), while total RNA from *T. b. gambiense* AnTat 22.1 was a gift from Stijn Deborggraeve and Veerle Lejon (Department of Parasitology, Institute of Tropical Medicine, Antwerp, Belgium). RNA samples were treated with DNase I (Ambion) and reverse-transcribed using Omniscript RT (Qiagen) and oligo dT. Spliced *TbDIP13* transcript was amplified from first-strand cDNA using primers to the *T. brucei* spliced leader sequence [61] and either the 3′ end of the DIP13 open reading frame or GFP C-terminal tag (primers SL-F, DIP-R3 and GFP-R1, Table S1). Amplified products were cloned into plasmid vector pCR2.1-TOPO (Invitrogen) and analysed by DNA sequencing (minimum of 10 clones per cell line).

### Knockdown of *T. brucei DIP13* by RNA Interference (RNAi)

The construct p2T7DIP13 was linearised with *Not*I, then transfected into mid-log phase *T. brucei* BSF strain Lister 427 and procyclic strain EATRO 1125 using the Nucleofector® system as described [60]. Stable transformants were selected by growth in 2.5 µg/ml (BSF) or 10 µg/ml (PCF) phleomycin. Production of dsRNA was induced in stable cell lines by incubating parasites in 1 µg/ml tetracycline.

### Production of *DIP13* knockout Lines

To generate *T. brucei DIP13* null lines (Δ*dip13*::*BLE*/Δ*dip13*::*PAC*), 5 µg of construct TbDIPKO-BLE was digested with *Sac*I/*Xba*I and transfected into BSF strain Lister 427 and PCF strain EATRO 1125 by nucleofection as above. Stable transformants were selected by growth in 2.5 µg/ml (BSF) or 10 µg/ml (PCF) phleomycin. Correct integration of the exogenous DNA fragment was confirmed by PCR amplification from genomic DNA of a 1067 bp product using primers BLE-KO-F (which anneals to the ACT 3′ UTR) and BLE-KO-R (which anneals to a region of the *DIP13* genomic locus downstream of the predicted integration site). Cells were then transfected with a second knockout construct, TbDIPKO-PAC, digested with *Sac*I/*Bsm*I. Stable transformants were selected by growth in phleomycin as above and 0.5 µg/ml (BSF) or 1 µg/ml (PCF) puromycin. Clonal populations were produced by two rounds of limiting dilution in 24-well plates. Correct integration of the puromycin resistance gene was confirmed by PCR amplification from genomic DNA of a 1015 bp product using primers PAC-KO-F (which anneals to the *ALD* 3′ UTR) and PAC-KO-R (which anneals to a region of the *DIP13* genomic locus downstream of the predicted integration site). Genomic DNA was analysed by Southern blotting using probes against the *DIP13* open reading frame, phleomycin resistance (*BLE*) and puromycin (*PAC*) resistance genes. Clones were also analysed for the presence of *DIP13* by quantitative Real-Time PCR of genomic DNA, as detailed below. Growth of confirmed *TbDIP13* null cell lines was monitored using a haemocytometer, diluting cultures to a concentration of 1×10^5^ cells/ml every 24 hours (BSF) or 1×10^6^/ml every 48 hours (PCF) and plotting cumulative numbers. Immunoblotting of total parasite lysates was performed as described [59].

### Quantitative Real-Time PCR

Absolute quantitation by qPCR was used to determine changes in *DIP13*-specific transcript following tetracycline induction of RNAi, relative to a constitutively expressed control, either α-tubulin or myristoyl-CoA:protein *N*-myristoyltransferase (NMT). Total RNA was extracted from parasites using Trizol reagent (Invitrogen) as described by the manufacturer. Traces of genomic DNA were removed by treatment with DNase I, prior to reverse transcription using Omniscript RT (Qiagen) and Oligo-dT (Promega). Alternatively, qPCR was used as a highly sensitive method to confirm the absence of *TbDIP13* in the genomic DNA of clonal populations of *DIP13* null parasite lines, compared to two other genes from the same chromosome (10): myristoyl-CoA:protein *N*-myristoyltransferase (*NMT*) and *ARL2*. The program Primer Express (Applied Biosystems) was used to design the following primers: DIP-qPCR-F, DIP-qPCR-R, NMT-qPCR-F, NMT-qPCR-R, ARL2-qPCR-F, ARL2-qPCR-R, αTUB-qPCR-F and αTUB-qPCR-R (Table S1). Quantitative PCR reactions were performed using SYBR Green Mastermix (Applied Biosystems) on an ABI 7000 Sequence Detection System (Applied Biosystems) and results analysed with Sequence Detection Software v1.2.3 (Applied Biosystems).

### Confocal Microscopy

Indirect immunofluorescence assays on parasites were performed as described [59]. Primary antibodies were used as follows: mouse monoclonal antibodies L13D6 against PFR1/2 and TAT1 against *T. brucei* α-tubulin (1∶50 and 1∶200 dilution respectively, gifts from Keith Gull, Sir William Dunn School of Pathology, University of Oxford, UK), mouse monoclonal 6-11B-1 against acetylated α-tubulin (1∶200, Sigma), rabbit anti-TbRab5A (1∶250, a gift from Mark Field, Department of Pathology, University of Cambridge, UK) and mouse anti-myc (1∶250, Invitrogen). Rabbit anti-TbDIP13 (described above) was used at 1∶20 dilution for immunofluorescence analysis. Primary antibodies were detected using Alexa Fluor 488 or 633 conjugated secondary antibodies (Invitrogen). Co-localization with rhodamine-labelled ConA (Sigma) was performed on ice or at 37°C for 10 minutes, using methods described previously [59]. Samples were visualized by confocal microscopy using a Zeiss LSM 510 meta with a Plan-Apochromat 63×/1.4 Oil DIC I objective lens and images acquired using LSM 510 version 3.2 software (Zeiss). Cell body (posterior end of the body to the flagellum tip) and PFR lengths (100 per sample) were measured from acquired images using LSM software as above. Statistical analysis (1 way ANOVA) was performed using GraphPad Prism 4.

### Electron Microscopy

Transmission electron microscopy of parasites was performed as described [62]. For scanning electron microscopy of intact parasites, 2×10^7^ log-phase cells (grown +/− tetracycline for 24 hours) were washed in PBS before settling onto poly L-lysine coated Thermanox coverslips (Nunc). After washing in PBS, cells were fixed in 1% gluteraldehyde for 1 hour then washed twice for 30 minutes in 100 mM phosphate buffer. All steps were performed in microcentrifuge tubes, briefly centrifuged and resuspended between each step. Cells were then dehydrated by suspending in an ethanol series of 50%, 70%, 90%, 100% for 30 minutes at each step, before addition of hexamethyl disilazane (HMDS) for 30 minutes. Cells were then air dried overnight. The pellet was vortexed, mounted on aluminium SEM stubs, coated with a thin layer (∼7 nm) of gold/palladium and visualised on a JEOL JSM-649OLV scanning electron microscope at 8 kV, spot size 35. For SEM of detergent-extracted cytoskeletons, cells were settled onto coverslips, then treated with 1% Triton-X/PBS at RT for 10 minutes, before fixing and processing as above.

For immuno-staining of cytoskeletons, cells were settled onto coverslips and detergent-extracted as above. Samples were incubated in blocking solution (1% fatty acid free BSA/PBS) for 30 minutes, then in primary antibody (rabbit anti-GFP, AbCam) diluted 1∶200 in blocking solution for 15 minutes. Coverslips were washed three times in blocking buffer, incubated in 4% paraformaldehyde for 20 minutes then washed three times in 20 mM glycine/PBS and once in blocking buffer. Samples were incubated in 10 nM gold conjugated goat anti-rabbit IgG (Agar Scientific) diluted 1∶10 in blocking buffer for 30 minutes then washed four times in blocking buffer. Cells were then fixed in 2.5% glutaraldehyde/PBS before dehydration and carbon coating as above. Samples were visualised on a JEOL JSM-7500F scanning electron microscope with backscattered electron detector.

For transmission electron microscopy of oligomerised protein, purified recombinant TbDIP13^His^ produced in *E. coli* (as described above, 10 mg/ml in 6 M urea) was diluted 1∶10 with 30 mM MOPS pH 7.0, incubated for 24 hours at 37°C, then diluted 1∶10 again to a final protein concentration of 0.1 mg/ml. A drop of suspension (8 µl) was placed on a 200 mesh copper grid with a Formvar/carbon support film for 2 minutes at room temperature, then stained with 1% uranyl acetate in water for 10 minutes. Viewing was at 120 kV with a Tecnai 12 BioTwin (FEI) and images captured with a SIS Megaview III digital camera.

### Immunoblotting with Human African Trypanosomiasis (HAT) patient sera

Immunoblots were prepared containing 30 ng of purified recombinant TbDIP13 protein (from *E. coli* as described above) or total lysate from 1×10^6^ *T. brucei* BSF Lister 427 parasites per lane. Blots were probed individually with 10 human serum samples collected in 2002–03 from Uganda [63], comprising 8 confirmed *T. b. rhodesiense* cases (including both early and late stages of the disease) and 2 uninfected controls. Blots were probed with sera diluted 1∶400 in blocking buffer (5% milk, 0.1% Tween-20 in PBS) then with horseradish peroxidase labelled goat anti human IgG (Sigma) diluted 1∶25,000 in blocking buffer. Signals were detected using ECL Plus reagents (GE Healthcare Life Sciences).

### Mouse Infections

Animal experiments were performed at the University of Cambridge in strict accordance with the UK Home Office Animal [Scientific Procedures] Act 1986, following approval by the University of Cambridge Ethical Review Panel, PPL number 80/2314. Swiss outbred (CD-1) mice were infected by i.p. injection with 2×10^5^ parasites of the *T. brucei* BSF parental Lister 427 or *DIP13* double knockout cell line. Parasitaemia was measured at 48 hours and 72 hours by microscopy analysis of tail-cut blood samples and the experiment terminated at the later timepoint.

### Comparative proteomics (iTRAQ) of flagellar extracts

Flagellar fractions were prepared from *T. brucei* BSF lines Lister 427 and lines stably transfected with pTbDIP13^GFP^ or pTbDIP13^myc^ following incubation in tetracycline for 24 hours. Protein samples were subjected to trypsin digestion and labelled using iTRAQ reagents (Applied Biosystems). Fractions were separated by Strong Cation Exchange chromatography and analysed by Nano-LC-ESI-MS. Full details are provided in Methods S1. Identified proteins and corresponding peptide sequences have been deposited with TriTrypDB (www.tritrypdb.org).

## Supporting Information

Figure S1 **Splice site analysis of** ***T. brucei DIP13*** **.** (A) Alignment of kinetoplastid DIP13 orthologues and related protein sequences. Sequence accession numbers (EMBL/TryTrypDB): Mouse, CAM14679.1; Human, O43805; *Schistosoma mansoni*, CAZ279931.1; *Chlamydomonas reinhardtii*, EDP00400.1; *Eimeria tenella*, CAK51393.1; *Plasmodium falciparum*, CAX64385.1; *Trypanosoma brucei gambiense*, Tbg972.10.16480; *Trypanosoma brucei brucei*, Tb10.61.2720 (now Tb927.10.14110); *Trypanosoma cruzi*, Tc00.1047053507993.369; *Leishmania major*, LmjF34.4540; *Leishmania infantum*, LinJ34_V3.4170; *Leishmania braziliensis*, LbrM20_V2.4000. (B) DNA sequence alignment of *T. b. brucei* and *T. b. gambiense DIP13* loci. The original annotated start codons for *T. b. brucei* and *T. b. gambiense* are shown in red and dark green boxes, respectively, and the stop codon in a grey box. The two annotated sequences differed by the apparent insertion of a single nucleotide (shown in an orange box) in *T. b. gambiense*. However, this insertion was found in some but not all of the *T. b. brucei* sequences. We performed splice site mapping by RT-PCR using primers to the splice leader sequence and the 3′ region of the *DIP13* ORF. The amplified products were cloned and DNA sequenced to find the position of the splice leader sequence within the *DIP13* gene. The trans-splicing acceptor site (AG) was mapped to 202 bases downstream of the annotated start codon in *T. b. brucei* and was in the same position in the *T. b. gambiense DIP13* sequence (marked by a light green box). We also found the annotated *T. b. gambiense* start codon (dark green box) to be ACG in all sequenced clones from both subspecies. This information was used to identify the correct start codons in the *T. b. brucei* and *T. b. gambiense* genes (dark blue box), both of which correspond to those of the other *DIP13* orthologues. (C) Sequence of the correct *T. brucei DIP13* ORF, with predicted coiled-coil shown in red text. (D, E) Expression of different ‘splice variants’ of *T. b. brucei* DIP13 fused to a C-terminal myc tag. Constructs encoding the *DIP13* ORF were generated in a myc tag vector, starting at the original annotated start codon (B above, red box) with no insertion at nucleotide position 76; the *T. b. gambiense* original annotated start codon (B, dark green box, sequence changed to ATG) or the elucidated start codon (B, dark blue box). Isoforms were named Long, Intermediate (Int) or Short, respectively. (D) Immunofluorescence of *T. b. brucei* BSF Lister 427 transfected with each of the three constructs and incubated in the presence of tetracycline for 24 hours. Cells were probed with mouse anti-myc and detected with Alexa Fluor 488 conjugated goat-anti-mouse (green). All cells were co-stained with DAPI (blue). Bar, 5 µm. (E) Total cell lysates (1×10^7^ cells/lane) from BSF parental line Lister 427 (wt) and cells as in D, grown in the presence of tetracycline for 24 hours, were immunoblotted and probed with mouse anti-myc and anti-BiP to monitor equal sample loading. L, Long isoform, I, Intermediate, S, Short isoform. The data show that the Long and Short isoforms produced proteins of the same size and subcellular localisation, corresponding to use of the elucidated endogenous splice acceptor site and correct start codon. The Intermediate isoform protein is slightly larger in size and has a different subcellular localisation, consistent with a short N-terminal extension but use of the same splice acceptor site as above. We conclude that the *DIP13* gene preferentially uses the splice acceptor site shown in B above, regardless of the presence of an upstream splice site in the expression vector. RT-PCR was also performed on material from BSF cells expressing the ‘Long’ isoform of DIP13 with a C-terminal GFP tag (data not shown). All clones utilised the endogenous splice acceptor site, supporting our findings for the myc-tagged isoforms.
(TIF)

Figure S2 **Detection of DIP13^GFP^ by scanning immuno-electron microscopy.** *T. brucei* BSF parasites of transgenic line 427/pTbDIP13GFP were incubated in the presence of tetracycline for 24 hours, then extracted with 1% Triton X-100, probed with rabbit anti-GFP (Abcam) and detected with 10 nm colloidal gold conjugated goat-anti-rabbit. Scanning electron micrographs are shown in (A) and (C) and immuno-electron micrographs of selected regions (marked by yellow boxes) are shown in (B) and (D). Areas containing gold particles are indicated by yellow arrows. Fl, flagellum. Bar, 1 µm (A, C) or 200 nm (B, D).
(TIF)

Figure S3 **Scanning electron micrographs of DIP13^GFP^-expressing cells.** (A, B) Scanning electron micrographs of intact *T. brucei* BSF lines Lister 427 (A) and 427/pTbDIP13GFP 24 hours post-induction (B). Both images show the region of the cell where the flagellum emerges from the flagellar pocket. (C, D) Scanning electron micrographs of BSF cells following extraction with 1% Triton X-100. (C) Lister 427 parental line, (D) 427/pTbDIP13GFP 24 hours post-induction, with an extraneous structure indicated by yellow arrows. Bars as shown.
(TIF)

Figure S4 **Comparative proteomics (iTRAQ) of** ***T. brucei*** **BSF flagellar extracts.** (A) Flagellar extracts (10 µg) from *T. brucei* BSF parental line Lister 427 (1) and transgenic BSF lines 427/ pTbDIP13GFP (2) and 427/ pTbDIP13myc (3) were separated by SDS-PAGE and stained with Sypro Ruby. Corresponding protein marker positions are shown (kDa). (B) Plot to show the reproducibility of iTRAQ ratio determination. The number of peptide events for each identified protein is plotted against the observed protein ratio for two experimental replicates of extracts from TbDIP13^myc^ expressing cells labelled with either 113 or 117 iTRAQ isobaric tags. Based on these data, proteins with less than 4 peptide events were excluded from further analysis (C) Plot as above to show the observed protein ratios for TbDIP13^myc^ expressing cells (113 tag) compared to parental control extract (114 tag).
(TIF)

Table S1 List of primer sequences.
(DOC)

Table S2 List of proteins identified in the flagellar extracts of *T. brucei* BSF cells with pI value less than 10.5.
(DOC)

Table S3 List of proteins identified in the flagellar extracts of *T. brucei* BSF cells with pI value greater than 10.5.
(DOC)

Table S4 List of proteins significantly enriched or depleted in the flagellar extracts of *T. brucei* BSF overexpressing DIP13 compared to the parental line.
(DOC)

Methods S1 Additional methods for comparative proteomic studies.
(DOC)

## References

[1] Fox RI (2005). Sjogren's syndrome.. Lancet.

[2] Ramos-Morales F, Infante C, Fedriani C, Bornens M, Rios RM (1998). NA14 is a novel nuclear autoantigen with a coiled-coil domain.. J Biol Chem.

[3] Nozawa K, Ikeda K, Satoh M, Reeves WH, Stewart CM (2009). Autoantibody to NA14 is an independent marker primarily for Sjogren's syndrome.. Front Biosci.

[4] Pfannenschmid F, Wimmer VC, Rios RM, Geimer S, Krockel U (2003). Chlamydomonas DIP13 and human NA14: a new class of proteins associated with microtubule structures is involved in cell division.. J Cell Sci.

[5] Andersen JS, Wilkinson CJ, Mayor T, Mortensen P, Nigg EA (2003). Proteomic characterization of the human centrosome by protein correlation profiling.. Nature.

[6] Aki T, Funakoshi T, Nishida-Kitayama J, Mizukami Y (2008). TPRA40/GPR175 regulates early mouse embryogenesis through functional membrane transport by Sjogren's syndrome-associated protein NA14.. J Cell Physiol.

[7] Lai CK, Gupta N, Wen X, Rangell L, Chih B (2011). Functional characterization of putative cilia genes by high-content analysis.. Mol Biol Cell.

[8] Errico A, Claudiani P, D'Addio M, Rugarli EI (2004). Spastin interacts with the centrosomal protein NA14, and is enriched in the spindle pole, the midbody and the distal axon.. Hum Mol Genet.

[9] Hazan J, Fonknechten N, Mavel D, Paternotte C, Samson D (1999). Spastin, a new AAA protein, is altered in the most frequent form of autosomal dominant spastic paraplegia.. Nat Genet.

[10] Schoppmeier J, Mages W, Lechtreck KF (2005). GFP as a tool for the analysis of proteins in the flagellar basal apparatus of Chlamydomonas.. Cell Motil Cytoskeleton.

[11] Mills E, Price HP, Johner A, Emerson JE, Smith DF (2007). Kinetoplastid PPEF phosphatases: dual acylated proteins expressed in the endomembrane system of Leishmania.. Mol Biochem Parasitol.

[12] Broadhead R, Dawe HR, Farr H, Griffiths S, Hart SR (2006). Flagellar motility is required for the viability of the bloodstream trypanosome.. Nature.

[13] Hart SR, Lau KW, Hao Z, Broadhead R, Portman N (2009). Analysis of the trypanosome flagellar proteome using a combined electron transfer/collisionally activated dissociation strategy.. J Am Soc Mass Spectrom.

[14] Bennett-Lovsey RM, Herbert AD, Sternberg MJ, Kelley LA (2008). Exploring the extremes of sequence/structure space with ensemble fold recognition in the program Phyre.. Proteins.

[15] Kelley LA, Sternberg MJ (2009). Protein structure prediction on the Web: a case study using the Phyre server.. Nat Protoc.

[16] Whitby FG, Phillips GN (2000). Crystal structure of tropomyosin at 7 Angstroms resolution.. Proteins.

[17] Hart D, Misset O, Edwards S, Opperdoes FR (1984). Comparison of the glycosomes (microbodies) isolated from *Trypanosoma brucei* blood stream form and cultured procyclic trypomastigotes.. Mol Biochem Parasitol.

[18] Lamour N, Riviere L, Coustou V, Coombs GH, Barrett MP (2005). Proline metabolism in procyclic Trypanosoma brucei is down-regulated in the presence of glucose.. J Biol Chem.

[19] Shapiro J, Ingram J, Johnson KA (2005). Characterization of a molecular chaperone present in the eukaryotic flagellum.. Eukaryot Cell.

[20] Bloch MA, Johnson KA (1995). Identification of a molecular chaperone in the eukaryotic flagellum and its localization to the site of microtubule assembly.. J Cell Sci.

[21] Guan J, Kinoshita M, Yuan L (2009). Spatiotemporal association of DNAJB13 with the annulus during mouse sperm flagellum development.. BMC Dev Biol.

[22] Affolter M, Hemphill A, Roditi I, Muller N, Seebeck T (1994). The repetitive microtubule-associated proteins MARP-1 and MARP-2 of Trypanosoma brucei.. J Struct Biol.

[23] Muller N, Hemphill A, Imboden M, Duvallet G, Dwinger RH (1992). Identification and characterization of two repetitive non-variable antigens from African trypanosomes which are recognized early during infection.. Parasitology.

[24] Muller N, Imboden M, Detmer E, Mansfield JM, Seebeck T (1993). Cytoskeleton-associated antigens from African trypanosomes are recognized by self-reactive antibodies of uninfected mice.. Parasitology.

[25] Jarvik JW, Telmer CA (1998). Epitope tagging.. Annu Rev Genet.

[26] Moutevelis E, Woolfson DN (2009). A periodic table of coiled-coil protein structures.. J Mol Biol.

[27] Wolf E, Kim PS, Berger B (1997). MultiCoil: a program for predicting two- and three-stranded coiled coils.. Protein Sci.

[28] McAlinden A, Smith TA, Sandell LJ, Ficheux D, Parry DA (2003). Alpha-helical coiled-coil oligomerization domains are almost ubiquitous in the collagen superfamily.. J Biol Chem.

[29] Chapman ER, An S, Barton N, Jahn R (1994). SNAP-25, a t-SNARE which binds to both syntaxin and synaptobrevin via domains that may form coiled coils.. J Biol Chem.

[30] Sheetz MP (1999). Motor and cargo interactions.. Eur J Biochem.

[31] Robson Marsden H, Kros A (2010). Self-assembly of coiled coils in synthetic biology: inspiration and progress.. Angew Chem Int Ed Engl.

[32] LeDizet M, Piperno G (1986). Cytoplasmic microtubules containing acetylated alpha-tubulin in Chlamydomonas reinhardtii: spatial arrangement and properties.. J Cell Biol.

[33] Piperno G, LeDizet M, Chang XJ (1987). Microtubules containing acetylated alpha-tubulin in mammalian cells in culture.. J Cell Biol.

[34] Sasse R, Gull K (1988). Tubulin post-translational modifications and the construction of microtubular organelles in Trypanosoma brucei.. J Cell Sci.

[35] Gibson W, Bailey M (1994). Genetic exchange in Trypanosoma brucei: evidence for meiosis from analysis of a cross between drug-resistant transformants.. Mol Biochem Parasitol.

[36] Gibson W, Peacock L, Ferris V, Williams K, Bailey M (2008). The use of yellow fluorescent hybrids to indicate mating in Trypanosoma brucei.. Parasit Vectors.

[37] Vickerman K (1985). Developmental cycles and biology of pathogenic trypanosomes.. Br Med Bull.

[38] Natesan SK, Peacock L, Matthews K, Gibson W, Field MC (2007). Activation of endocytosis as an adaptation to the mammalian host by trypanosomes.. Eukaryot Cell.

[39] Lacomble S, Portman N, Gull K (2009). A protein-protein interaction map of the Trypanosoma brucei paraflagellar rod.. PLoS One.

[40] El-Sayed NM, Myler PJ, Bartholomeu DC, Nilsson D, Aggarwal G (2005). The genome sequence of Trypanosoma cruzi, etiologic agent of Chagas disease.. Science.

[41] Casanova M, Crobu L, Blaineau C, Bourgeois N, Bastien P (2009). Microtubule-severing proteins are involved in flagellar length control and mitosis in Trypanosomatids.. Mol Microbiol.

[42] Nozawa K, Fritzler MJ, von Muhlen CA, Chan EK (2004). Giantin is the major Golgi autoantigen in human anti-Golgi complex sera.. Arthritis Res Ther.

[43] Mack GJ, Rees J, Sandblom O, Balczon R, Fritzler MJ (1998). Autoantibodies to a group of centrosomal proteins in human autoimmune sera reactive with the centrosome.. Arthritis Rheum.

[44] Scofield RH (2004). Autoantibodies as predictors of disease.. Lancet.

[45] Bizzaro N (2007). Autoantibodies as predictors of disease: the clinical and experimental evidence.. Autoimmun Rev.

[46] Mor F, Weinberger A, Cohen IR (2002). Identification of alpha-tropomyosin as a target self-antigen in Behcet's syndrome.. Eur J Immunol.

[47] Reese G, Ayuso R, Lehrer SB (1999). Tropomyosin: an invertebrate pan-allergen.. Int Arch Allergy Immunol.

[48] Selkoe DJ (2004). Cell biology of protein misfolding: the examples of Alzheimer's and Parkinson's diseases.. Nat Cell Biol.

[49] Eichner T, Radford SE (2011). Understanding the complex mechanisms of beta2-microglobulin amyloid assembly.. FEBS J.

[50] Haass C, Selkoe DJ (2007). Soluble protein oligomers in neurodegeneration: lessons from the Alzheimer's amyloid beta–peptide.. Nat Rev Mol Cell Biol.

[51] Rosenberg AS (2006). Effects of protein aggregates: an immunologic perspective.. AAPS J.

[52] Hertz-Fowler C, Peacock CS, Wood V, Aslett M, Kerhornou A (2004). GeneDB: a resource for prokaryotic and eukaryotic organisms.. Nucleic Acids Res.

[53] Aslett M, Aurrecoechea C, Berriman M, Brestelli J, Brunk BP (2010). TriTrypDB: a functional genomic resource for the Trypanosomatidae.. Nucleic Acids Res.

[54] McNicholas S, Potterton E, Wilson KS, Noble ME (2011). Presenting your structures: the CCP4mg molecular-graphics software.. Acta Crystallogr D Biol Crystallogr.

[55] Emsley P, Lohkamp B, Scott WG, Cowtan K (2010). Features and development of Coot.. Acta Crystallogr D Biol Crystallogr.

[56] Wirtz E, Leal S, Ochatt C, Cross GA (1999). Trypanosoma brucei variant surface glycoprotein regulation involves coupled activation/inactivation and chromatin remodeling of expression sites.. Mol Biochem Parasitol.

[57] Brun R, Jenni L (1977). A new semi-defined medium for Trypanosoma brucei sspp.. Acta Trop.

[58] Alsford S, Kawahara T, Isamah C, Horn D (2007). A sirtuin in the African trypanosome is involved in both DNA repair and telomeric gene silencing but is not required for antigenic variation.. Mol Microbiol.

[59] Price HP, Panethymitaki C, Goulding D, Smith DF (2005). Functional analysis of TbARL1, an N-myristoylated Golgi protein essential for viability in bloodstream trypanosomes.. J Cell Sci.

[60] Burkard G, Fragoso CM, Roditi I (2007). Highly efficient stable transformation of bloodstream forms of Trypanosoma brucei.. Mol Biochem Parasitol.

[61] Boothroyd JC, Cross GA (1982). Transcripts coding for variant surface glycoproteins of Trypanosoma brucei have a short, identical exon at their 5′ end.. Gene.

[62] Price HP, Stark M, Smith B, Smith DF (2007). TbARF1 influences lysosomal function but not endocytosis in procyclic stage Trypanosoma brucei.. Mol Biochem Parasitol.

[63] Maclean L, Odiit M, Macleod A, Morrison L, Sweeney L (2007). Spatially and genetically distinct African Trypanosome virulence variants defined by host interferon-gamma response.. J Infect Dis.

