# Supplementary figures and images for: The Orthologue of Sjögren's Syndrome Nuclear Autoantigen 1 (SSNA1) in Trypanosoma brucei Is an Immunogenic Self-Assembling Molecule
Source: PLoS One. 2012 Feb 20;7(2):e31842. doi: 10.1371/journal.pone.0031842 (PMC3282761; doi:10.1371/journal.pone.0031842)

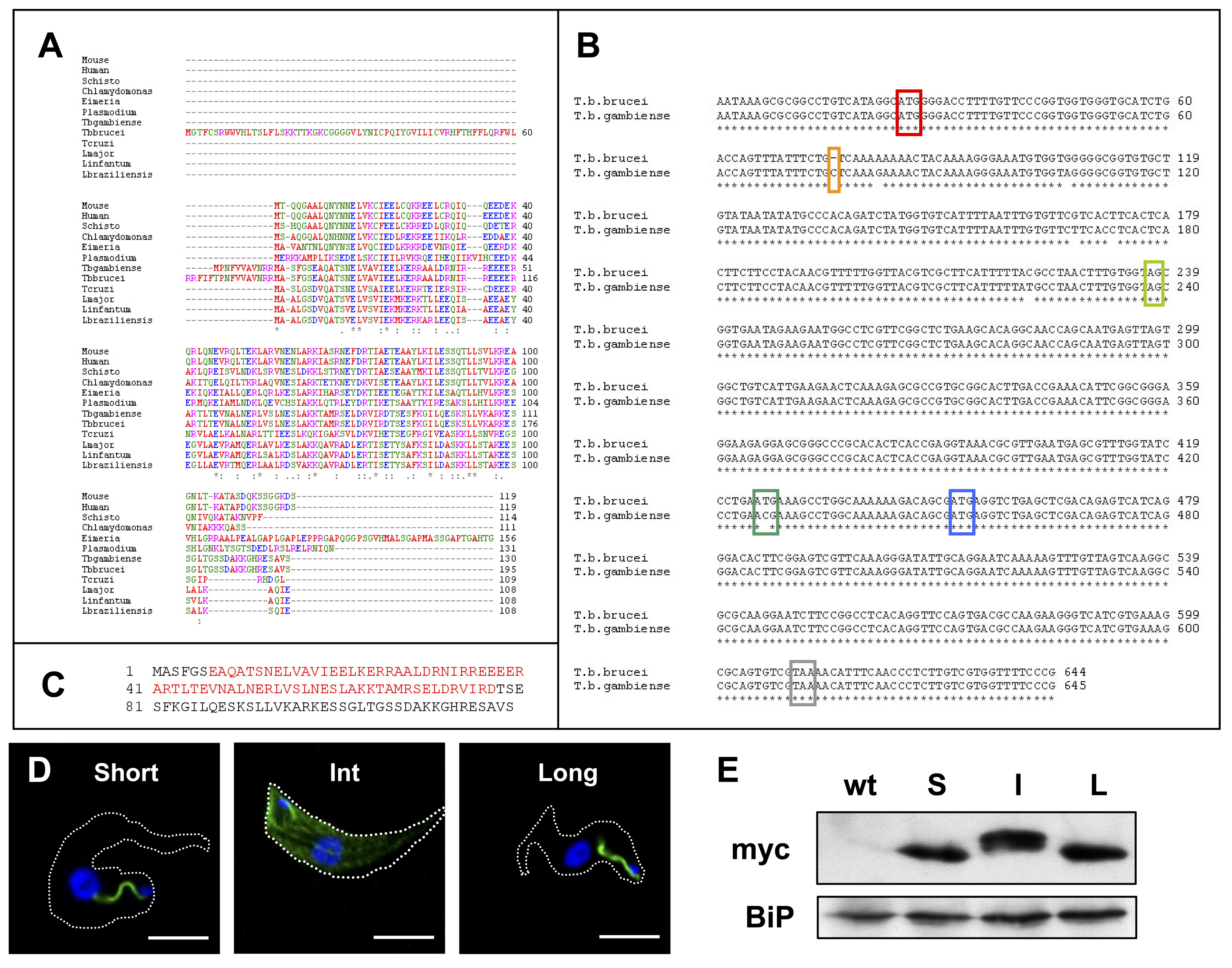

Supplement: Figure S1 — Splice site analysis of T. brucei DIP13 . (A) Alignment of kinetoplastid DIP13 orthologues and related protein sequences. Sequence accession numbers (EMBL/TryTrypDB): Mouse, CAM14679.1; Human, O43805; Schistosoma mansoni, CAZ279931.1; Chlamydomonas reinhardtii, EDP00400.1; Eimeria tenella, CAK51393.1; Plasmodium falciparum, CAX64385.1; Trypanosoma brucei gambiense, Tbg972.10.16480; Trypanosoma brucei brucei, Tb10.61.2720 (now Tb927.10.14110); Trypanosoma cruzi, Tc00.1047053507993.369; Leishmania major, LmjF34.4540; Leishmania infantum, LinJ34_V3.4170; Leishmania braziliensis, LbrM20_V2.4000. (B) DNA sequence alignment of T. b. brucei and T. b. gambiense DIP13 loci. The original annotated start codons for T. b. brucei and T. b. gambiense are shown in red and dark green boxes, respectively, and the stop codon in a grey box. The two annotated sequences differed by the apparent insertion of a single nucleotide (shown in an orange box) in T. b. gambiense. However, this insertion was found in some but not all of the T. b. brucei sequences. We performed splice site mapping by RT-PCR using primers to the splice leader sequence and the 3′ region of the DIP13 ORF. The amplified products were cloned and DNA sequenced to find the position of the splice leader sequence within the DIP13 gene. The trans-splicing acceptor site (AG) was mapped to 202 bases downstream of the annotated start codon in T. b. brucei and was in the same position in the T. b. gambiense DIP13 sequence (marked by a light green box). We also found the annotated T. b. gambiense start codon (dark green box) to be ACG in all sequenced clones from both subspecies. This information was used to identify the correct start codons in the T. b. brucei and T. b. gambiense genes (dark blue box), both of which correspond to those of the other DIP13 orthologues. (C) Sequence of the correct T. brucei DIP13 ORF, with predicted coiled-coil shown in red text. (D, E) Expression of different ‘splice variants’ of T. b. brucei DIP [file pone.0031842.s001.tif]

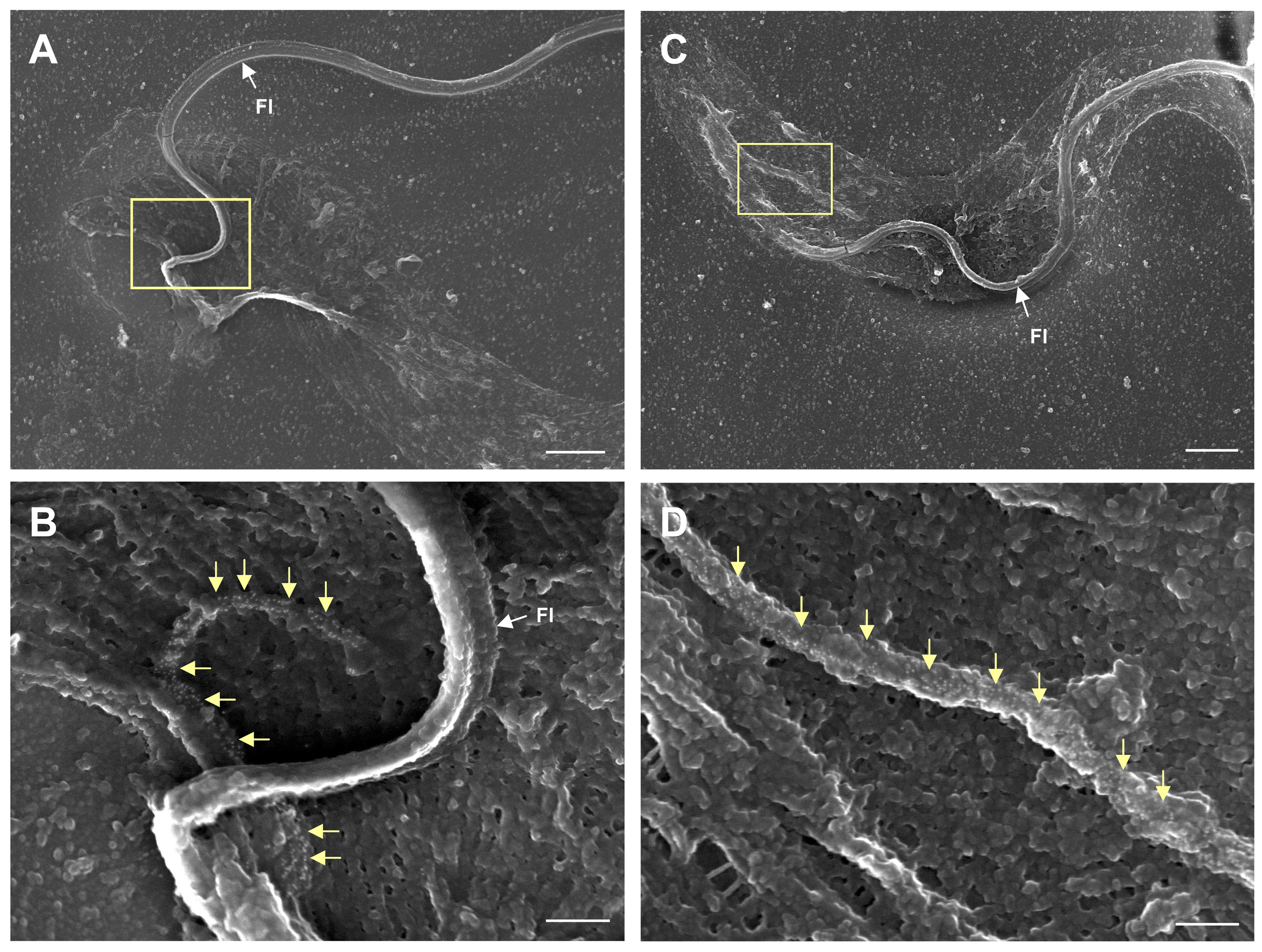

Supplement: Figure S2 — Detection of DIP13GFP by scanning immuno-electron microscopy. T. brucei BSF parasites of transgenic line 427/pTbDIP13GFP were incubated in the presence of tetracycline for 24 hours, then extracted with 1% Triton X-100, probed with rabbit anti-GFP (Abcam) and detected with 10 nm colloidal gold conjugated goat-anti-rabbit. Scanning electron micrographs are shown in (A) and (C) and immuno-electron micrographs of selected regions (marked by yellow boxes) are shown in (B) and (D). Areas containing gold particles are indicated by yellow arrows. Fl, flagellum. Bar, 1 µm (A, C) or 200 nm (B, D). (TIF) [file pone.0031842.s002.tif]

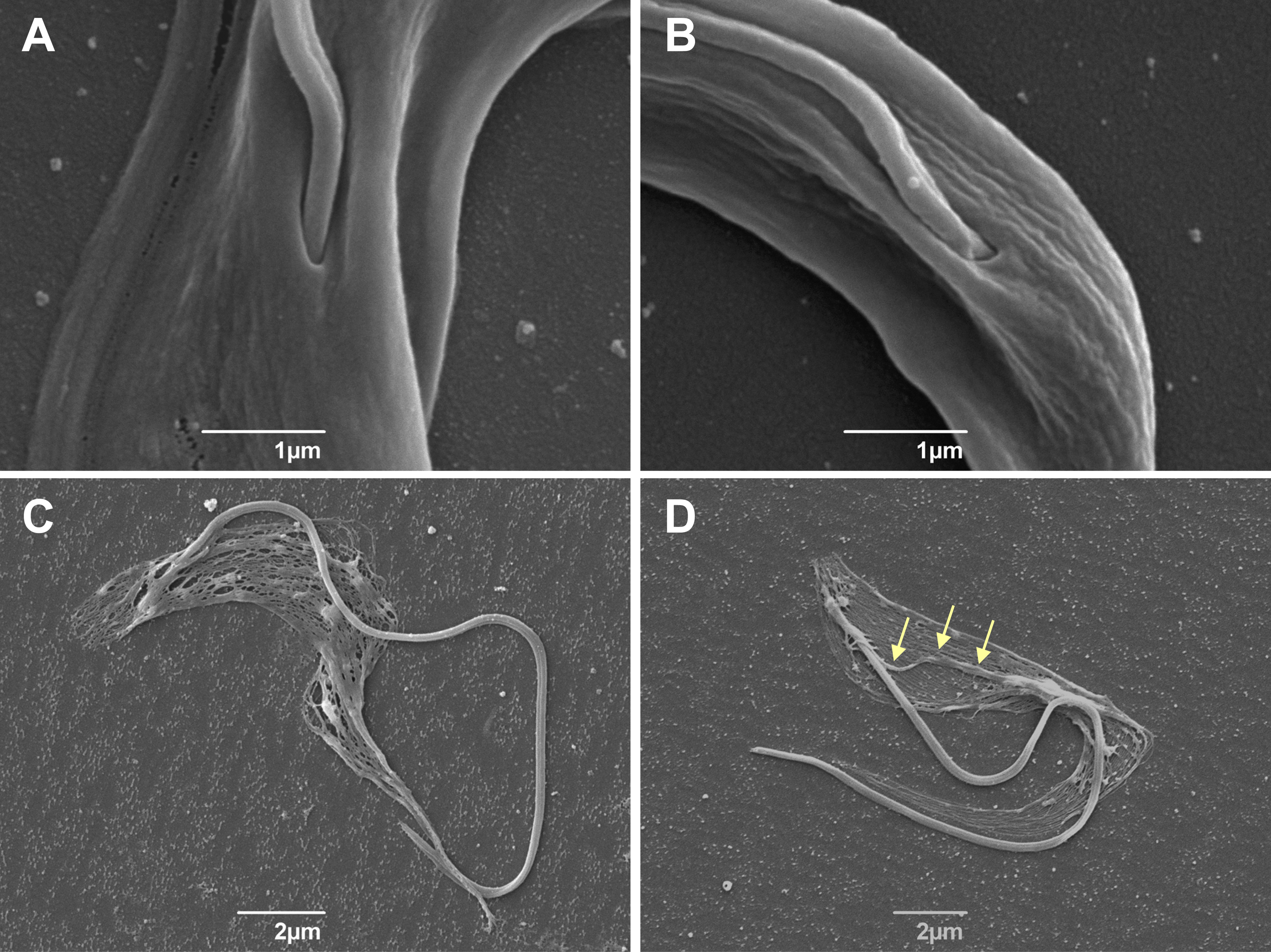

Supplement: Figure S3 — Scanning electron micrographs of DIP13GFP-expressing cells. (A, B) Scanning electron micrographs of intact T. brucei BSF lines Lister 427 (A) and 427/pTbDIP13GFP 24 hours post-induction (B). Both images show the region of the cell where the flagellum emerges from the flagellar pocket. (C, D) Scanning electron micrographs of BSF cells following extraction with 1% Triton X-100. (C) Lister 427 parental line, (D) 427/pTbDIP13GFP 24 hours post-induction, with an extraneous structure indicated by yellow arrows. Bars as shown. (TIF) [file pone.0031842.s003.tif]

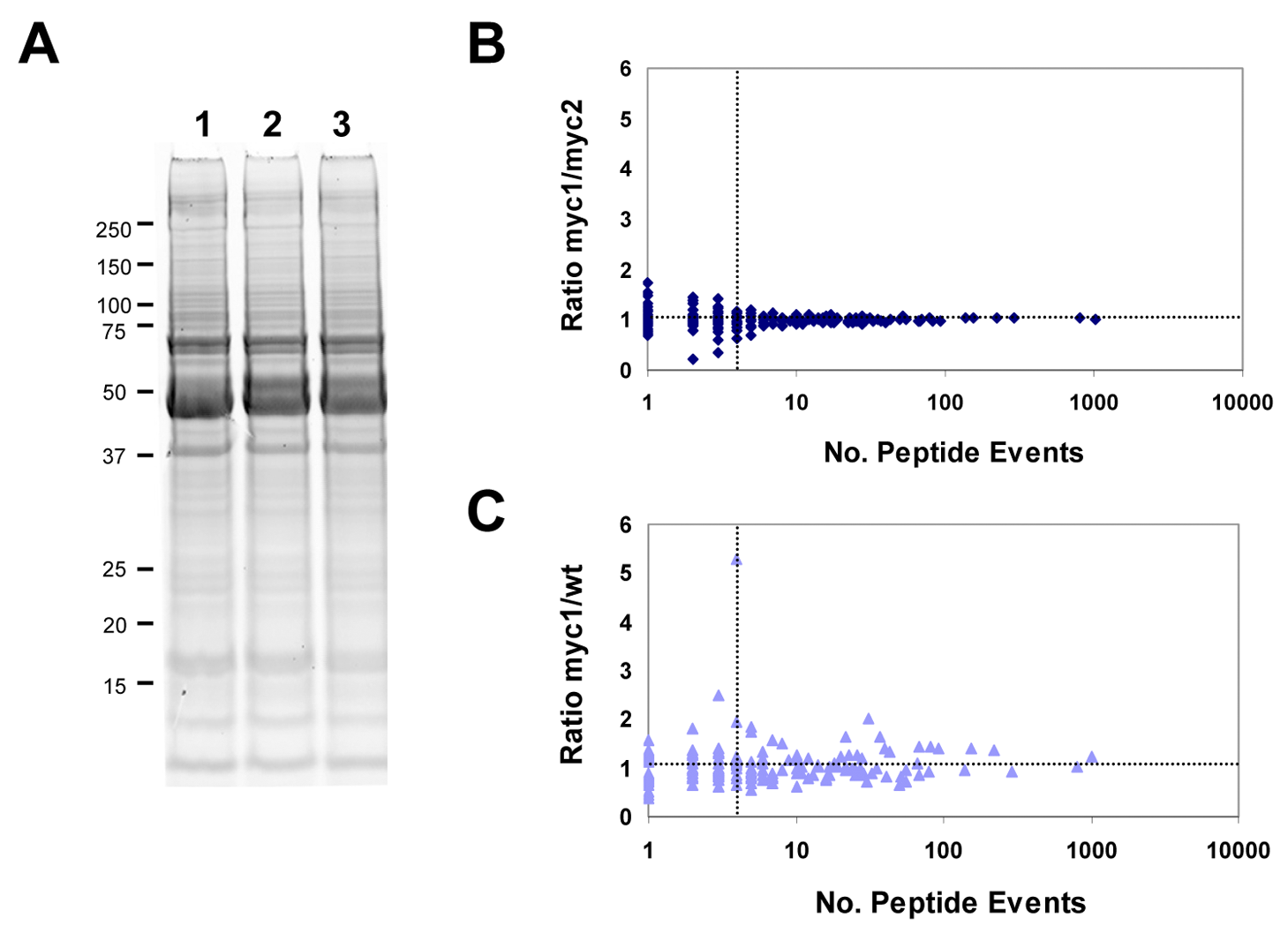

Supplement: Figure S4 — Comparative proteomics (iTRAQ) of T. brucei BSF flagellar extracts. (A) Flagellar extracts (10 µg) from T. brucei BSF parental line Lister 427 (1) and transgenic BSF lines 427/ pTbDIP13GFP (2) and 427/ pTbDIP13myc (3) were separated by SDS-PAGE and stained with Sypro Ruby. Corresponding protein marker positions are shown (kDa). (B) Plot to show the reproducibility of iTRAQ ratio determination. The number of peptide events for each identified protein is plotted against the observed protein ratio for two experimental replicates of extracts from TbDIP13myc expressing cells labelled with either 113 or 117 iTRAQ isobaric tags. Based on these data, proteins with less than 4 peptide events were excluded from further analysis (C) Plot as above to show the observed protein ratios for TbDIP13myc expressing cells (113 tag) compared to parental control extract (114 tag). (TIF) [file pone.0031842.s004.tif]
