# Supplementary material for: The Orthologue of Sjögren's Syndrome Nuclear Autoantigen 1 (SSNA1) in Trypanosoma brucei Is an Immunogenic Self-Assembling Molecule
Source: PLoS One. 2012 Feb 20;7(2):e31842. doi: 10.1371/journal.pone.0031842 (PMC3282761; doi:10.1371/journal.pone.0031842)
Supplement: Table S1 — List of primer sequences. (DOC) [file pone.0031842.s005.doc]

| **Primer** | **Sequence** |
| --- | --- |
| DIP-F1 | 5’-GTTATGGGGACCTTTTGTTCCCGGTGGTG-3’ |
| DIP-R1 | 5’-ACGACACTGCGCTTTCACGATGACCCTT-3’ |
| DIP-F2 | 5’-GATCCACTAGTCCAGTGTGGTGGAATTG-3’ |
| DIP-R2 | 5’-CGTGCAACTAGAAGGCACAGTCGA-3’ |
| DIP-F3 | 5’-AATAAAGCTTGGCCTGTCATAGGCATGGG-3’ |
| DIP-R3 | 5’-GACAAGAGGGTTGATCTAGACGACACTGCG-3’ |
| DIP-F4 | 5’-ACGAAGCTTCATTTTTATGCCTAACTTTG-3’ |
| DIP-F5 | 5’-TACGCCTAAGCTTGTGGTAGCGGTGAATAG-3’ |
| DIP-F6 | 5’-CATCGTGAAAGCGCAGTGTCGTAAAGAGAAGAGAAAC-3’ |
| DIP-R6 | 5’-ACGACACTGCGCTTTCACGATGACCCTTCTTGG-3’ |
| DIP-F7 | 5’-TCGTTCGGCTCTAGAGCACAGGCAACCAGC-3’ |
| DIP-R7 | 5’-CGCTTTCTAGATGACCCTTCTTGGCGT-3’ |
| DIP-F8 | 5’-GCACTTAACTTCTCGAGTTTGCCTTATAGTTTTCCC-3’ |
| DIP-R8 | 5’-CAAGTTAACTAACGGCTCTAGATGTGAGTGGTCG-3’ |
| DIP-F9 | 5’-CAACAAAATGGAGCTCTTGCGCCGGGTATC-3’ |
| DIP-R9 | 5’-GATAATAATGAGATATCCGTCAACACAGTCCCCTTCCC-3’ |
| DIP-F10 | 5’-CACCATGGCCTCGTTCGGCTCTGAAG-3’ |
| DIP-R10 | 5’-CGACACTGCGCTTTCACGATG-3’ |
| BLE-F | 5’-AAAGATATCGCCCGGGCACAGCAAGGTC-3’ |
| BLE-R | 5’-GGGCTCGAGAATACTGCATAGATAACAAACGCATCAA-3’ |
| PAC-F | 5’-GAAGATATCAATAGTGCCACCAGTTGTGTTTG-3’ |
| PAC-R | 5’-ATTCTCGAGGCTACAATTAATACATAACC-3’ |
| SL-F | 5’-CGCTATTATTAGAACAGTTTCTGTAC-3’ |
| GFP-R1 | 5’-GGGTAAGCTTTCCGTATGTAGC-3’ |
| BLE-KO-F | 5’-TGAGTTGACACGGCTAGTGC-3’ |
| BLE-KO-R | 5’-ATTTCCGTGTCGCTGAAGTT-3’ |
| PAC-KO-F | 5’-TTCCCGGAGTAATCTGATGG-3’ |
| PAC-KO-R | 5’-ATTTCCGTGTCGCTGAAGTT-3’ |
| DIP-qPCR-F | 5’-TTCGGCGGGAGGAAGAG-3’ |
| DIP-qPCR-R | 5’-GGGATACCAAACGCTCATTCA-3’ |
| NMT-qPCR-F | 5’-CGAAAGCATGTTTCGATTCAAC-3’ |
| NMT-qPCR-R | 5’-GGTGATATCCCGGAGGCATA-3’ |
| ARL2-qPCR-F | 5’-ACGTTGCTCGTTTGGATGACT-3’ |
| ARL2-qPCR-R | 5’-AGCCGTTCTTCCTGTAATAGTGTGT-3’ |
| αTUB-qPCR-F | 5’-ATGCGTGAGGCTATCTGCAT-3’ |
| αTUB-qPCR-R | 5’-CTAGTACTCCTCCACATCCTCCTCA-3’ |
