# Supplementary material for: The Orthologue of Sjögren's Syndrome Nuclear Autoantigen 1 (SSNA1) in Trypanosoma brucei Is an Immunogenic Self-Assembling Molecule
Source: PLoS One. 2012 Feb 20;7(2):e31842. doi: 10.1371/journal.pone.0031842 (PMC3282761; doi:10.1371/journal.pone.0031842)
Supplement: Table S2 — List of proteins identified in the flagellar extracts of T. brucei BSF cells with pI value less than 10.5. (DOC) [file pone.0031842.s006.doc]

| **Accession No.** | **#Peptide Events** | **#Non-duplicate peptides** | **% Cov** | **Seq Tag** | **GeneDB Annotation** | **pI** | **MW** | **PCF Flagellum*** |
| --- | --- | --- | --- | --- | --- | --- | --- | --- |
| Tb09.160.1160 | 26 | 8 | 14.4 | MNRSC | Hypothetical protein, conserved | 4.4 | 85.9 | + |
| Tb09.160.1200 | 7 | 4 | 39.8 | MMQER | GB4 mitotubule-associated protein Gb4, putative; dynein heavy chain, cytosolic, putative | nd | 928.3 | + |
| Tb09.160.1660 | 1 | 1 | 19.8 | MSFGL | Hypothetical protein, conserved | 9.3 | 9.2 | + |
| Tb09.160.2070 | 2 | 1 | 5.3 | MAIVG | Cyclophilin type peptidyl-prolyl cis-trans isomerase, putative | 5.3 | 35.7 | + |
| Tb09.160.2360 | 5 | 2 | 10.9 | MFPFY | Poly(A) export protein, putative | 7.9 | 38.3 | + |
| Tb09.160.3590 | 1 | 1 | 1.1 | MFMNK | PDE2C cAMP-specific phosphodiesterase | 5.7 | 103.6 | + |
| Tb09.160.3960 | 1 | 1 | 2.9 | MRIPE | Actin, putative | 6.2 | 46.7 | + |
| Tb09.160.4280 | 4 | 3 | 18.6 | MSCGD | TRYP1 tryparedoxin peroxidase | 6.4 | 22.4 | + |
| Tb09.211.0630 | 3 | 1 | 5.6 | MSDEE | Actin A | 5.3 | 41.8 | + |
| Tb09.211.1370 | 6 | 2 | 11.3 | MEAET | Glyceraldehyde-3-phosphate dehydrogenase, putative | 5.7 | 38.8 | + |
| Tb09.211.1470 | 5 | 1 | 4.2 | MAFSR | Hypothetical protein, conserved | 10.1 | 35.3 | + |
| Tb09.211.2150 | 7 | 3 | 9.2 | MAAFA | Poly(A)-binding protein 1; PABP2 | 10.1 | 62.1 | - |
| Tb09.211.3550 | 50 | 12 | 27.3 | MKYVG | glk1 glycerol kinase, glycosomal | 8.2 | 56.3 | - |
| Tb09.211.4513 | 17 | 8 | 48.9 | MATTY | Kinetoplastid membrane protein KMP-11 | 6.5 | 11.0 | - |
| Tb09.211.4920 | 1 | 1 | 22.4 | MSEIE | Dynein-associated protein, putative | 6.5 | 11.1 | + |
| Tb10.26.0070 | 1 | 1 | 4.3 | MGLLK | 33 kDa inner dynein arm light chain, axonemal, putative; dynein light chain, putative | 7.9 | 42.0 | + |
| Tb10.26.0680 | 10 | 5 | 42.1 | MSHYL | Hypothetical protein, conserved | 5.6 | 14.3 | + |
| Tb10.26.0760 | 1 | 1 | 1.7 | MSDVP | Hypothetical protein, conserved | 5.3 | 101.1 | + |
| Tb10.389.0890 | 4 | 1 | 7.4 | MLKCV | Tb927.3.1790 | 7.5 | 42.5 | - |
| Tb10.389.1320 | 1 | 1 | 5.3 | MEVKI | Hypothetical protein, conserved | 5.1 | 24.6 | + |
| Tb10.406.0560 | 82 | 8 | 68.9 | MPGTK | Microtubule-associated protein, putative | 5.2 | 237.4 | + |
| Tb10.406.0640 | 3 | 3 | 28.6 | MHTGD | Hypothetical protein, conserved | 10.1 | 20.1 | - |
| Tb10.61.0150 | 1 | 1 | 2.7 | MDTVT | Inosine-5'-monophosphate dehydrogenase; IMP dehydrogenase | 9.0 | 48.4 | - |
| Tb10.61.0540 | 66 | 3 | 14.3 | MKSEG | Hypothetical protein, conserved | 4.7 | 36.4 | + |
| Tb10.61.0560 | 2 | 1 | 1.9 | MSGLT | Hypothetical protein, conserved | 7.3 | 197.7 | + |
| Tb10.61.0940 | 2 | 1 | 4.9 | MKNAL | Hypothetical protein, conserved | 6.4 | 32.7 | + |
| Tb10.61.1550 | 5 | 3 | 13.7 | MIGGQ | Hypothetical protein, conserved | 7.3 | 48.0 | + |
| Tb10.61.1630 | 1 | 1 | 4.9 | MYGNQ | RNA binding protein, putative | 7.5 | 46.1 | - |
| Tb10.61.1750 | 2 | 1 | 2.3 | MSVEQ | TBKIFC1 C-terminal motor kinesin, putative | 7.3 | 90.8 | - |
| Tb10.61.2130 | 1 | 1 | 3.3 | MLTVC | ATP-dependent DEAD/H RNA helicase, putative | 10.1 | 71.3 | - |
| Tb10.61.2210 | 27 | 7 | 30.0 | MSVKL | Hypothetical protein, conserved | 7.6 | 37.0 | + |
| Tb10.61.2220 | 24 | 6 | 23.8 | MLREQ | Hypothetical protein, conserved | 7.7 | 46.2 | + |
| **Tb10.61.2720** | **4** | **1** | **5.6** | **MGTFC** | **Hypothetical protein, conserved (DIP13)** | **8.0** | **13.2** | **+** |
| Tb10.61.2870 | 4 | 2 | 5.4 | MTYQA | Tb10.61.2870 hypothetical protein, conserved | 4.6 | 50.6 | + |
| Tb10.6k15.0140 | 30 | 4 | 31.7 | MYRSR | Hypothetical protein, conserved | 5.2 | 37.0 | - |
| Tb10.6k15.0810 | 28 | 6 | 11.9 | MEKTQ | Hypothetical protein, conserved; leucine-rich repeat protein (LRRP), putative | 4.9 | 118.6 | + |
| Tb10.6k15.1500 | 22 | 7 | 31.1 | MSATV | Hypothetical protein, conserved | 9.2 | 45.3 | + |
| Tb10.6k15.1510 | 2 | 2 | 5.6 | MDCVY | Hypothetical protein, conserved | 5.0 | 80.0 | + |
| Tb10.6k15.1760 | 5 | 1 | 0.9 | MKAAQ | Hypothetical protein, conserved | 4.6 | 244.8 | + |
| Tb10.6k15.3460 | 3 | 1 | 0.6 | MNTSF | Hypothetical protein, conserved | 5.2 | 284.7 | - |
| Tb10.70.0430 | 55 | 13 | 40.9 | MFRCV | HSP60 chaperonin Hsp60, mitochondrial precursor | 5.1 | 59.5 | + |
| Tb10.70.1370 | 154 | 20 | 58.6 | MSKRV | ALD fructose-bisphosphate aldolase, glycosomal, putative | 9.0 | 41.0 | - |
| Tb10.70.1720 | 7 | 1 | 0.4 | MSISY | Dynein heavy chain, putative | 5.8 | 474.7 | + |
| Tb10.70.3290 | 5 | 1 | 4.2 | MVTDD | DHH1 ATP-dependent DEAD-box RNA helicase, putative; DHH1 | 7.5 | 46.4 | - |
| Tb10.70.5440 | 1 | 1 | 4.3 | MVKET | Chaperone protein DNAJ, putative | 6.9 | 46.7 | - |
| Tb10.70.5560 | 7 | 3 | 13.3 | MFHNS | Hypothetical protein, conserved | 7.1 | 34.5 | + |
| Tb10.70.5670 | 16 | 5 | 20.9 | MGKEK | TEF1 elongation factor 1-alpha; EF-1-alpha | 9.4 | 49.1 | + |
| Tb10.70.6570 | 1 | 1 | 5.6 | MDGPF | Hypothetical protein, conserved | 4.3 | 329.6 | + |
| Tb10.70.7560 | 3 | 2 | 17.3 | MPRYY | Hypothetical protein, conserved | 4.7 | 25.5 | + |
| Tb11.01.0510 | 2 | 1 | 2.9 | MEEGT | Hypothetical protein, conserved | 6.8 | 46.4 | + |
| Tb11.01.1210 | 1 | 1 | 3.8 | MNELK | Hypothetical protein, conserved | 6.8 | 61.7 | + |
| Tb11.01.1475 | 4 | 2 | 26.7 | MGFFD | 40S ribosomal protein S27 | 10.1 | 9.6 | - |
| Tb11.01.1625 | 25 | 4 | 61.2 | MQQSL | Hypothetical protein, conserved | 4.4 | 11.8 | - |
| Tb11.01.1680 | 3 | 1 | 17.1 | MQIFV | Polyubiquitin, putative | 7.9 | 76.6 | + |
| Tb11.01.1850 | 1 | 1 | 2.9 | MSSPM | Hypothetical protein, conserved | 8.6 | 46.3 | + |
| Tb11.01.2310 | 23 | 1 | 3.3 | MRVKR | Hypothetical protein, conserved | 5.5 | 99.4 | + |
| Tb11.01.2460 | 1 | 1 | 4.4 | MKRQR | Hypothetical protein, conserved | 9.1 | 45.2 | - |
| Tb11.01.2680 | 2 | 1 | 10.8 | MTETS | 40S ribosomal protein SA | 8.2 | 31.3 | - |
| Tb11.01.2800 | 35 | 9 | 36.8 | MPLPP | Hypothetical protein, conserved | 10.1 | 41.6 | + |
| Tb11.01.3000 | 10 | 2 | 9.4 | MKTKF | Hypothetical protein, conserved | 5.2 | 41.9 | - |
| Tb11.01.3010 | 1 | 1 | 1.1 | MGTGA | Dynein heavy chain, putative | 6.0 | 480.2 | + |
| Tb11.01.3110 | 28 | 7 | 21.2 | MTYEG | Heat shock protein 70 | 6.3 | 75.3 | + |
| Tb11.01.3550 | 2 | 1 | 5.2 | MLRRL | 2-oxoglutarate dehydrogenase, E2 component, dihydrolipoamide succinyltransferase, putative | 8.1 | 41.1 | + |
| Tb11.01.3960 | 20 | 4 | 10.7 | MAFLV | Hypothetical protein, conserved; calmodulin-like protein containing EF hand domain (BILBO1) | 5.7 | 67.3 | + |
| Tb11.01.4390 | 4 | 1 | 2.2 | MQQLA | Leucine-rich repeat protein (LRRP), putative | 5.1 | 70.0 | + |
| Tb11.01.4624 | 79 | 7 | 76.5 | MADQL | Calmodulin | 3.9 | 16.8 | + |
| Tb11.01.4810 | 3 | 1 | 9.7 | MRSKT | Hypothetical protein, conserved | 9.6 | 23.9 | + |
| Tb11.01.5100 | 55 | 9 | 21.6 | MIEVQ | Paraflagellar rod component, putative | 5.2 | 68.3 | + |
| Tb11.01.5800 | 1 | 1 | 1.4 | MLTIP | Calpain-like cysteine peptidase, putative; cysteine peptidase, Clan CA, family C2, putative | 6.9 | 165.6 | + |
| Tb11.01.6510 | 3 | 3 | 8.9 | MSADP | Hypothetical protein, conserved | 3.8 | 41.0 | + |
| Tb11.01.6740 | 8 | 4 | 3.6 | MAVVH | Hypothetical protein, conserved | 3.8 | 127.9 | + |
| Tb11.01.6780 | 10 | 3 | 24.1 | MADLY | Chaperone protein DNAJ, putative; heat shock protein-like protein, putative | 5.2 | 35.3 | + |
| Tb11.01.8770 | 28 | 8 | 16.3 | MSASA | Hypothetical protein, conserved; leucine-rich repeat protein (LRRP), putative | 4.3 | 110.1 | + |
| Tb11.02.0210 | 37 | 4 | 25.8 | MAAAV | Hypothetical protein, conserved | 4.9 | 50.8 | + |
| Tb11.02.0760 | 7 | 2 | 1.1 | MAKGD | Dynein heavy chain, putative | 5.2 | 531.1 | + |
| Tb11.02.0990 | 3 | 2 | 3.6 | MSVEE | Hypothetical protein, conserved | 4.8 | 115.1 | + |
| Tb11.02.2060 | 6 | 1 | 9.2 | MPVNP | Flagellar radial spoke component, putative; flagellar radial spoke protein 4, putative | 4.0 | 66.9 | + |
| Tb11.02.2210 | 1 | 1 | 3.4 | MSEKG | PKA-R protein kinase A regulatory subunit | 5.1 | 56.7 | - |
| Tb11.02.3200 | 3 | 1 | 6.4 | MPTRP | Dynein arm light chain, axonemal, putative | 5.6 | 27.3 | + |
| Tb11.02.3210 | 1 | 1 | 6.0 | MSKPQ | TIM triosephosphate isomerase | 9.5 | 26.8 | - |
| Tb11.02.4320 | 25 | 4 | 11.8 | MSLVG | Hypothetical protein, conserved | 6.9 | 107.3 | + |
| Tb11.02.4380 | 1 | 1 | 2.9 | MFGGS | Hypothetical protein, conserved | 9.1 | 45.8 | + |
| Tb11.02.5280 | 4 | 3 | 9.3 | MSRIP | Glycerol-3-phosphate dehydrogenase, putative | 8.1 | 66.9 | - |
| Tb11.02.5500 | 33 | 6 | 20.5 | MSRMW | Glucose-regulated protein 78, putative; luminal binding protein 1 (BiP), putative | 5.2 | 71.4 | - |
| Tb11.03.0140 | 4 | 2 | 2.8 | MSAGF | Nucleoporin, putative; serine peptidase, Clan SP, family S59, putative | 7.5 | 158.3 | + |
| Tb11.03.0230 | 4 | 1 | 4.6 | MNKIS | IDH isocitrate dehydrogenase, putative | 8.1 | 46.7 | - |
| Tb11.03.0815 | 1 | 1 | 20.0 | MMSDR | Dynein light chain, putative | 8.4 | 10.4 | - |
| Tb11.39.0004 | 3 | 1 | 6.8 | METAE | Hypothetical protein, conserved | 5.7 | 45.6 | + |
| Tb11.46.0002 | 1 | 1 | 8.6 | MPSVS | 60S acidic ribosomal subunit protein | 4.9 | 34.6 | - |
| Tb11.47.0006 | 28 | 9 | 24.7 | MAYQQ | Hypothetical protein, conserved | 5.9 | 86.8 | + |
| Tb11.47.0034 | 4 | 2 | 14.2 | MQGQN | Radial spoke protein 3, putative; radial spoke 3 protein, putative | 6.0 | 39.3 | + |
| Tb11.50.0001 | 5 | 3 | 18.6 | MELME | Hypothetical protein, conserved | 9.4 | 30.1 | + |
| Tb11.57.0008 | 10 | 3 | 23.6 | MACQE | Calpain-like protein, putative; cytoskeleton assocociated protein, putative | nd | nd | + |
| Tb927.1.120 | 2 | 2 | 2.5 | MNQQF | Retrotransposon hot spot (RHS) protein, putative; retrotransposon hot spot protein 4 (RHS4), putative | 8.9 | 98.0 | - |
| Tb927.1.2230 | 1 | 1 | 8.9 | MGCGG | Calpain-like protein fragment | 5.8 | 13.5 | + |
| Tb927.1.2390 | 807 | 24 | 57.7 | MREIV | Beta tubulin | 4.5 | 49.7 | + |
| Tb927.1.2400 | 1013 | 24 | 66.5 | MREAI | Alpha tubulin | 4.7 | 49.7 | + |
| Tb927.1.2670 | 6 | 1 | 3.7 | MPNRQ | Axoneme central apparatus protein, putative; importin alpha-1 subunit, putative | 6.6 | 56.1 | + |
| Tb927.1.3830 | 1 | 1 | 6.9 | MSNYL | PGI glucose-6-phosphate isomerase, glycosomal | 7.5 | 67.6 | - |
| Tb927.1.4310 | 11 | 5 | 7.6 | MENDY | Hypothetical protein, conserved | 9.7 | 183.7 | + |
| Tb927.1.700 | 5 | 2 | 9.3 | MTLNE | PGKC phosphoglycerate kinase | 9.9 | 47.2 | - |
| Tb927.2.2160 | 13 | 5 | 21.6 | MTSEE | Hypothetical protein, conserved | 5.0 | 37.7 | - |
| Tb927.2.2770 | 1 | 1 | 7.2 | MPVIV | Hypothetical protein, conserved | 5.8 | 13.4 | - |
| Tb927.2.4230 | 41 | 5 | 22.9 | MFSAG | NUP-1 protein, putative | 4.7 | 406.8 | + |
| Tb927.2.5660 | 8 | 4 | 24.2 | MAVLS | Adenylate kinase, putative | 5.7 | 29.3 | + |
| Tb927.2.5760 | 3 | 1 | 3.3 | MGEAD | Hypothetical protein, conserved | 6.3 | 330.0 | - |
| Tb927.3.1120 | 1 | 1 | 16.6 | MQASS | rtb2 GTP-binding nuclear protein rtb2, putative | 7.4 | 24.4 | + |
| Tb927.3.1790 | 6 | 2 | 8.3 | MRRFA | Pyruvate dehydrogenase E1 beta subunit, putative | 5.2 | 37.5 | - |
| Tb927.3.2230 | 1 | 1 | 6.6 | MLSRT | Succinyl-CoA synthetase alpha subunit, putative | 8.6 | 31.4 | - |
| Tb927.3.2310 | 2 | 1 | 6.0 | MSYEI | Hypothetical protein, conserved | 9.9 | 33.7 | + |
| Tb927.3.3180 | 2 | 2 | 3.3 | MRERP | Hypothetical protein, conserved | 6.9 | 98.1 | + |
| Tb927.3.3270 | 69 | 15 | 33.7 | MAVES | TbPFK ATP-dependent phosphofructokinase | 9.7 | 53.5 | - |
| Tb927.3.3750 | 14 | 6 | 63.1 | MSTQL | Hypothetical protein, conserved | 5.3 | 19.8 | + |
| Tb927.3.3790 | 16 | 4 | 24.8 | MSATE | Hypothetical protein, conserved | 4.4 | 31.6 | - |
| Tb927.3.4330 | 292 | 31 | 57.4 | MAAVD | PFR1 73 kDa paraflagellar rod protein; PFR1 | 5.9 | 68.6 | + |
| Tb927.3.4720 | 4 | 2 | 5.8 | MERLI | Dynamin, putative | 6.9 | 73.3 | - |
| Tb927.3.5350 | 2 | 1 | 18.1 | MPARA | Hypothetical protein, conserved | 9.2 | 11.5 | - |
| Tb927.3.930 | 5 | 1 | 0.5 | MKAVQ | Dynein heavy chain, putative | 6.4 | 531.4 | + |
| Tb927.4.1300 | 31 | 4 | 22.2 | MSTAL | Hypothetical protein, conserved | 6.2 | 42.0 | + |
| Tb927.4.2070 | 29 | 6 | 46.6 | MEILE | Antigenic protein, putative | 4.1 | 511.3 | + |
| Tb927.4.2080 | 18 | 4 | 8.4 | MQQVD | Hypothetical protein, conserved | 5.7 | 104.8 | + |
| Tb927.4.2260 | 2 | 1 | 11.0 | MSSSR | Centrin, putative | 4.3 | 21.1 | + |
| Tb927.4.3740 | 19 | 3 | 23.2 | MALLN | Hypothetical protein, conserved | 4.3 | 192.5 | - |
| Tb927.4.4040 | 3 | 1 | 6.6 | MDNTE | Hypothetical protein, conserved | 4.5 | 30.2 | - |
| Tb927.4.4690 | 2 | 2 | 10.4 | MIGGA | Hypothetical protein, conserved | 9.3 | 31.3 | + |
| Tb927.4.4700 | 2 | 1 | 6.3 | MAQTS | Hypothetical protein, conserved | 9.1 | 30.7 | + |
| Tb927.5.2080 | 1 | 1 | 4.9 | MSFNE | Inosine-5'-monophosphate dehydrogenase, putative | 9.6 | 52.2 | - |
| Tb927.5.4390 | 1 | 1 | 14.8 | MSGVV | Hypothetical protein, conserved | 5.2 | 15.0 | - |
| Tb927.6.3800 | 12 | 4 | 19.0 | MLARR | Heat shock 70 kDa protein, mitochondrial precursor, putative | 5.8 | 71.4 | - |
| Tb927.6.3820 | 1 | 1 | 1.8 | MESSV | Hypothetical protein, conserved | 6.5 | 93.6 | + |
| Tb927.6.4140 | 5 | 1 | 33.0 | MPNLV | Hypothetical protein, conserved | 6.5 | 13.0 | + |
| Tb927.6.4300 | 220 | 11 | 51.5 | MTIKV | GAPDH glyceraldehyde 3-phosphate dehydrogenase, glycosomal | 9.6 | 43.8 | + |
| Tb927.6.4520 | 3 | 1 | 4.1 | MDAGD | Hypothetical protein, conserved | 8.1 | 52.5 | + |
| Tb927.6.4670 | 22 | 5 | 22.1 | MIYSG | Hypothetical protein, conserved | 5.1 | 40.6 | + |
| Tb927.6.5070 | 9 | 3 | 13.0 | MRANP | Hypothetical protein, conserved | 6.1 | 51.8 | + |
| Tb927.7.1920 | 2 | 1 | 2.2 | MLQAA | Leucine-rich repeat protein (LRRP), putative | 4.9 | 59.2 | + |
| Tb927.7.2650 | 12 | 6 | 11.0 | MSTTA | Hypothetical protein, conserved | 5.9 | 62.1 | - |
| Tb927.7.3330 | 52 | 7 | 38.3 | MATAE | Hypothetical protein, conserved | 4.2 | 502.6 | + |
| Tb927.7.3410 | 6 | 1 | 9.4 | MAALT | Centrin, putative | 4.1 | 16.5 | + |
| Tb927.7.3440 | 2 | 1 | 43.9 | MLCPP | I/6 autoantigen | 4.4 | 27.0 | + |
| Tb927.7.3550 | 93 | 5 | 7.4 | MSIFG | Hypothetical protein, conserved | 5.3 | 138.1 | + |
| Tb927.7.3740 | 10 | 1 | 6.1 | MSYVV | Hypothetical protein, conserved | 4.5 | 93.2 | + |
| Tb927.7.4100 | 3 | 1 | 3.0 | MATEV | Hypothetical protein, conserved | 8.4 | 56.7 | + |
| Tb927.7.6910 | 11 | 5 | 8.5 | MSGSD | Hypothetical protein, conserved | 4.6 | 92.2 | + |
| Tb927.8.1550 | 28 | 9 | 23.0 | MSATY | Hypothetical protein, conserved | 5.5 | 88.1 | + |
| Tb927.8.2070 | 3 | 1 | 8.0 | MGCGG | Hypothetical protein, conserved | 5.1 | 21.7 | - |
| Tb927.8.3060 | 1 | 1 | 2.2 | MLKRV | Cytosolic leucyl aminopeptidase, putative | 9.7 | 71.3 | - |
| Tb927.8.3530 | 43 | 7 | 30.2 | MVSGV | Glycerol-3-phosphate dehydrogenase [NAD+], glycosomal | 8.8 | 37.8 | + |
| Tb927.8.3750 | 1 | 1 | 2.5 | MSKTL | Nucleolar protein, putative | 8.9 | 54.3 | - |
| Tb927.8.3790 | 5 | 1 | 11.7 | MDAEE | Hypothetical protein, conserved | 5.8 | 25.1 | + |
| Tb927.8.4580 | 14 | 4 | 9.3 | MIGMM | Hypothetical protein, conserved | 7.5 | 58.0 | + |
| Tb927.8.4780 | 17 | 2 | 4.2 | MRRRN | Hypothetical protein, conserved | 5.0 | 468.1 | + |
| Tb927.8.5010 | 138 | 31 | 69.3 | MSGKE | PFR2 69 kDa paraflagellar rod protein | 5.8 | 69.5 | + |
| Tb927.8.6230 | 3 | 1 | 4.3 | MVRWG | Hypothetical protein, conserved | 9.6 | 37.2 | + |
| Tb927.8.6240 | 22 | 6 | 38.0 | MTTLH | Hypothetical protein, conserved | 8.9 | 30.0 | + |
| Tb927.8.6660 | 68 | 11 | 27.5 | MTTMQ | Hypothetical protein, conserved | 5.2 | 69.0 | + |
| Tb927.8.6920 | 2 | 1 | 6.4 | MTKGG | Hypothetical protein, conserved | 4.5 | 29.5 | + |
| Tb927.8.810 | 5 | 3 | 21.0 | MSSVS | Hypothetical protein, conserved | 4.3 | 37.5 | + |
| Tb927.8.8330 | 40 | 9 | 17.3 | MGCGG | Calpain, putative | 4.2 | 98.4 | + |
| ) | | | | | | | | |
