# Supplementary material for: The Orthologue of Sjögren's Syndrome Nuclear Autoantigen 1 (SSNA1) in Trypanosoma brucei Is an Immunogenic Self-Assembling Molecule
Source: PLoS One. 2012 Feb 20;7(2):e31842. doi: 10.1371/journal.pone.0031842 (PMC3282761; doi:10.1371/journal.pone.0031842)
Supplement: Table S3 — List of proteins identified in the flagellar extracts of T. brucei BSF cells with pI value greater than 10.5. (DOC) [file pone.0031842.s007.doc]

| **Accession No.** | **#Peptide Events** | **#Non-duplicate peptides** | **% Cov** | **Seq Tag** | **GeneDB Annotation** | **pI** | **MW** | **PCF Flagellum*** |
| --- | --- | --- | --- | --- | --- | --- | --- | --- |
| Tb09.160.2550 | 3 | 2 | 12.4 | MSAQP | Ribosomal protein S7 | 11.9 | 23.8 | - |
| Tb09.160.4450 | 8 | 6 | 30.1 | MFLFS | 40S ribosomal protein S3 | 10.2 | 30.4 | + |
| Tb09.160.5590 | 1 | 1 | 7.2 | MAEAK | 60S ribosomal protein L11 | 11.0 | 22.3 | - |
| Tb09.211.0340 | 7 | 3 | 24.9 | MARRP | QM 60S ribosomal protein L10; QM-like protein | 11.2 | 24.7 | + |
| Tb09.211.2640 | 9 | 4 | 31.7 | MGKDK | 60S ribosomal protein L23 | 11.0 | 14.9 | + |
| Tb09.211.2730 | 3 | 3 | 20.2 | MSAQA | gim5A Gim5A protein; glycosomal membrane protein | 10.2 | 26.4 | - |
| Tb09.244.2630 | 1 | 1 | 6.4 | MKLNI | 40S ribosomal protein S6 | 12.0 | 28.4 | + |
| Tb09.244.2725 | 2 | 1 | 12.8 | MAATK | Ribosomal protein L36 | 12.1 | 12.4 | - |
| Tb09.244.2740 | 1 | 1 | 2.9 | MTFVK | 60S ribosomal protein L5 | 10.9 | 34.6 | - |
| Tb10.100.0155 | 2 | 1 | 19.7 | MVKPF | 60S ribosomal protein L32 | 11.9 | 15.3 | - |
| Tb10.26.0560 | 4 | 2 | 17.7 | MPAAT | 60S ribosomal protein L6 | 11.1 | 21.1 | - |
| Tb10.406.0460 | 15 | 3 | 29.5 | MATPK | Histone H2B | 12.2 | 12.5 | - |
| Tb10.61.1260 | 7 | 4 | 10.3 | MASST | Hypothetical protein, conserved | 10.4 | 57.8 | - |
| Tb10.61.2070 | 15 | 5 | 33.1 | MADAP | 40S ribosomal protein S2 | 10.8 | 28.6 | + |
| Tb10.6k15.3350 | 1 | 1 | 9.5 | MVFQK | 40S ribosomal protein S24E | 11.7 | 15.6 | - |
| Tb10.70.0465 | 1 | 1 | 19.4 | MAKRT | 60S ribosomal proteins L37 | 11.8 | 10.4 | - |
| Tb10.70.1540 | 23 | 2 | 18.4 | MRTID | 60S ribosomal protein L24 | 12.0 | 14.6 | + |
| Tb10.70.1740 | 1 | 1 | 15.7 | MSLTL | 40S ribosomal protein S18 | 11.4 | 17.5 | - |
| Tb10.70.3360 | 4 | 2 | 13.3 | MTLGK | 40S ribosomal protein S3a | 10.8 | 29.4 | + |
| Tb10.70.7010 | 2 | 1 | 11.6 | MKIKS | 60S ribosomal protein L9 | 10.7 | 21.8 | + |
| Tb10.70.7695 | 12 | 2 | 13.2 | MTSKQ | 40S ribosomal proteins S11 | 11.5 | 20.0 | - |
| Tb11.01.0355 | 2 | 1 | 15.3 | MTTKR | Ribosomal protein S26 | 11.9 | 12.8 | - |
| Tb11.01.1470 | 2 | 2 | 7.9 | MSKIP | 60S ribosomal protein L10a | 10.3 | 24.5 | - |
| Tb11.01.1480 | 1 | 1 | 4.7 | MSCPR | 60S ribosomal protein L34 | 12.5 | 19.3 | + |
| Tb11.01.1960 | 4 | 1 | 3.5 | MKSKD | Hypothetical protein, conserved | 10.8 | 53.4 | - |
| Tb11.01.3676 | 1 | 1 | 14.1 | MGKIR | 40S ribosomal protein S17 | 11.5 | 16.2 | - |
| Tb11.01.3805 | 5 | 2 | 28.9 | MDRRL | CAP15 microtubule-associated protein; corset-associated protein 15 | 10.4 | 14.0 | - |
| Tb11.01.4030 | 3 | 1 | 12.4 | MFSEP | Hypothetical protein, conserved | 10.3 | 32.4 | + |
| Tb11.01.5720 | 35 | 2 | 18.4 | MVRPH | Ribosomal protein L18 | 11.2 | 20.9 | - |
| Tb11.01.7545 | 7 | 2 | 8.3 | MKFLK | 60S ribosomal protein L27 | 12.3 | 15.5 | - |
| Tb11.02.1090 | 19 | 3 | 11.0 | MAKKH | 40S ribosomal protein S4 | 11.1 | 30.6 | + |
| Tb11.02.1840 | 13 | 3 | 19.7 | MGVDL | 60S ribosomal protein L18 | 12.3 | 21.8 | - |
| Tb11.02.2430 | 3 | 1 | 9.0 | MVHYS | 60S ribosomal protein L17 | 11.6 | 19.1 | - |
| Tb11.02.4000 | 2 | 1 | 10.8 | MTMMS | 40S ribosomal protein S15a | 10.7 | 14.6 | - |
| Tb11.02.4170 | 2 | 1 | 7.9 | MSAKA | 40S ribosomal protein S5 | 11.0 | 21.3 | - |
| Tb11.02.4350 | 1 | 1 | 7.7 | MATIG | 40S ribosomal protein S21 | 11.8 | 21.2 | - |
| Tb11.47.0026 | 4 | 2 | 7.1 | MVTTQ | Hypothetical protein, conserved | 10.5 | 53.3 | - |
| Tb927.1.2550 | 16 | 5 | 45.9 | MSRTK | Histone H3 | 11.6 | 14.7 | - |
| Tb927.2.2670 | 2 | 1 | 19.0 | MAKGK | Histone H4 | 11.7 | 11.2 | - |
| Tb927.2.5910 | 1 | 1 | 6.0 | MVRMH | 40S ribosomal protein S13 | 11.4 | 17.3 | + |
| Tb927.3.3300 | 6 | 4 | 9.2 | MATLN | Hypothetical protein, conserved | 10.4 | 89.1 | - |
| Tb927.3.3310 | 2 | 1 | 11.4 | MLFIA | 60S ribosomal protein L13 | 12.0 | 26.6 | + |
| Tb927.3.5050 | 15 | 5 | 25.7 | MTARP | 60S ribosomal protein L4 | 12.1 | 41.8 | - |
| Tb927.4.1100 | 14 | 3 | 22.0 | MVHSH | Ribosomal protein L21E (60S) | 11.1 | 18.0 | - |
| Tb927.4.1790 | 15 | 3 | 15.6 | MLTLL | Ribosomal protein L3 | 11.7 | 54.4 | - |
| Tb927.4.1860 | 1 | 1 | 9.0 | MAVAR | Ribosomal protein S19 | 11.3 | 18.8 | - |
| Tb927.4.2180 | 1 | 1 | 10.1 | MALVK | 60S ribosomal protein L35A | 12.1 | 17.0 | + |
| Tb927.4.3550 | 10 | 2 | 6.7 | MRVCV | 60S ribosomal protein L13a | 12.1 | 39.1 | + |
| Tb927.5.1110 | 4 | 1 | 6.5 | MGKTV | 60S ribosomal protein L2; 60S ribosomal protein L8 | 11.3 | 28.3 | - |
| Tb927.5.4260 | 25 | 6 | 44.0 | MAKGK | Histone H4 | 11.6 | 11.1 | + |
| Tb927.6.1470 | 2 | 1 | 4.0 | MRRDD | Hypothetical protein, conserved | 12.2 | 27.5 | - |
| Tb927.6.4980 | 16 | 3 | 34.7 | MSKKQ | 40S ribosomal protein S14 | 10.5 | 15.5 | + |
| Tb927.6.5040 | 1 | 1 | 7.4 | MGAFM | Ribosomal protein L15 | 12.5 | 24.4 | - |
| Tb927.7.1050 | 13 | 1 | 18.1 | MSGEK | 40S ribosomal protein S16 | 10.9 | 16.9 | - |
| Tb927.7.1750 | 2 | 1 | 6.6 | MFMTG | Ribosomal protein L7 | 11.2 | 29.3 | - |
| Tb927.7.2940 | 6 | 1 | 16.4 | MATPK | Histone H2A | 11.8 | 14.2 | - |
| Tb927.7.5180 | 3 | 1 | 6.8 | MMISF | 60S ribosomal protein L23a | 11.4 | 24.7 | - |
| Tb927.7.6360 | 3 | 1 | 5.0 | MSLTG | Histone H2A | 11.2 | 18.6 | - |
| Tb927.8.1110 | 3 | 2 | 11.1 | MRNYN | 40S ribosomal protein S9 | 11.2 | 22.0 | + |
| Tb927.8.1340 | 4 | 2 | 10.1 | MAGKE | 60S ribosomal protein L7a | 11.6 | 30.8 | + |
| Tb927.8.6030 | 1 | 1 | 9.1 | MPPKF | 60S ribosomal protein L12 | 10.7 | 17.5 | - |
| Tb927.8.6160 | 2 | 1 | 11.4 | MGIVR | 40S ribosomal protein S8 | 11.6 | 24.9 | - |
