# Supplementary material for: The Orthologue of Sjögren's Syndrome Nuclear Autoantigen 1 (SSNA1) in Trypanosoma brucei Is an Immunogenic Self-Assembling Molecule
Source: PLoS One. 2012 Feb 20;7(2):e31842. doi: 10.1371/journal.pone.0031842 (PMC3282761; doi:10.1371/journal.pone.0031842)
Supplement: Table S4 — List of proteins significantly enriched or depleted in the flagellar extracts of T. brucei BSF overexpressing DIP13 compared to the parental line. (DOC) [file pone.0031842.s008.doc]

| **ACC. NO.** | **NAME** | **MW** | **pI** | **FEATURES** | **GFP/WT** | **PVal** | **G2A/WT** | **PVal** | **Myc1/WT** | **PVal** | **Myc2/WT** | **PVal** |
| --- | --- | --- | --- | --- | --- | --- | --- | --- | --- | --- | --- | --- |
| Tb10.61.2720 | Hypothetical protein (DIP13) | 22.1 | 10.7 | Coiled coil, transmembrane domain | **4.253** | **0.000** | **3.767** | **0.000** | **5.767** | **0.000** | **5.292** | **0.000** |
| Tb927.2.5660 | Adenylate kinase | 29.3 | 5.7 | ADK, zinc finger lid region | **1.917** | **0.049** | 1.150 | 0.487 | 1.324 | 0.223 | 1.495 | 0.119 |
| Tb11.50.0001 | Hypothetical protein | 30.1 | 9.4 | Unknown | **1.478** | **0.000** | 1.158 | 0.210 | **1.474** | **0.016** | **1.736** | **0.010** |
| Tb927.7.3410 | Centrin | 16.5 | 4.1 | EF-hands domain | **1.477** | **0.004** | **1.401** | **0.032** | 1.263 | 0.102 | **1.327** | **0.012** |
| Tb927.3.3750 | Hypothetical protein | 19.8 | 5.3 | GPI anchored | **1.352** | **0.001** | **1.308** | **0.008** | 1.092 | 0.214 | 1.042 | 0.495 |
| Tb10.6k15.1500 | Hypothetical protein | 45.0 | 9.2 | Unknown | **1.263** | **0.002** | **1.132** | **0.026** | **1.580** | **0.000** | **1.626** | **0.000** |
| Tb10.61.2210 | Hypothetical protein | 37.0 | 7.6 | Unknown | **1.239** | **0.006** | **1.173** | **0.004** | 0.999 | 0.983 | 0.986 | 0.714 |
| Tb927.1.2400 | Alpha tubulin | 49.7 | 4.7 | Tubulin, coiled coil, GTP-binding, EEY/F$ motif | **1.196** | **0.000** | **1.087** | **0.000** | **1.242** | **0.000** | **1.231** | **0.000** |
| Tb10.406.0560 | Microtubule-associated protein | 237.4 | 5.2 | Repeats | **1.167** | **0.000** | **0.862** | **0.003** | **1.420** | **0.000** | **1.437** | **0.000** |
| Tb927.6.4300 | GAPDH | 43.8 | 9.6 | GAPDH, Rossmann NAD(P) binding folds | **0.899** | **0.011** | 1.028 | 0.359 | **1.410** | **0.000** | **1.353** | **0.000** |
| Tb927.1.4310 | Hypothetical protein | 183.7 | 9.7 | Coiled coil, PDZ domain | **0.835** | **0.036** | **0.825** | **0.001** | 0.919 | 0.234 | 0.901 | 0.161 |
| Tb927.1.2390 | Beta tubulin | 49.7 | 4.5 | Tubulin, coiled coil, GTP-binding | **0.829** | **0.000** | **0.912** | **0.000** | **1.057** | **0.000** | 1.023 | 0.131 |
| Tb927.2.2160 | Hypothetical protein | 37.7 | 5.0 | NSF attachment protein | **0.825** | **0.002** | 0.984 | 0.825 | **0.824** | **0.001** | **0.853** | **0.035** |
| Tb10.70.1370 | Aldolase | .41.0 | 9.0 | Aldolase-type TIM barrel, paxillin LD motif, PTS2 pattern | **0.816** | **0.000** | **0.928** | **0.003** | **1.458** | **0.000** | **1.395** | **0.000** |
| Tb927.3.3790 | Hypothetical protein | 31.6 | 4.4 | EF-hands domain | **0.794** | **0.006** | **0.884** | **0.011** | **0.766** | **0.000** | **0.759** | **0.000** |
| Tb11.02.5500 | Glucose-regulated protein 78 (BiP) | 71.4 | 5.2 | ATP-binding | **0.790** | **0.047** | 0.935 | 0.432 | **0.864** | **0.037** | 0.878 | 0.061 |
| Tb927.4.2070 | Antigenic protein | 511.3 | 4.1 | Coiled coil, repeats | **0.789** | **0.001** | **0.738** | **0.000** | **0.889** | **0.001** | **0.892** | **0.001** |
| Tb927.8.3530 | Glycerol-3-phosphate dehydrogenase [NAD+] | 37.8 | 8.8 | NAD-Gly3P-DH, NAD(P) binding, PTS2 pattern | **0.770** | **0.000** | **0.821** | **0.002** | **1.343** | **0.000** | **1.318** | **0.000** |
| Tb10.70.0430 | HSP60 chaperonin | 59.5 | 5.1 | ATP-binding | **0.741** | **0.001** | **1.180** | **0.006** | **0.703** | **0.000** | **0.708** | **0.000** |
| Tb927.2.4230 | NUP-1 protein | 408.0 | 4.7 | Coiled coil, repeats | **0.731** | **0.000** | **0.840** | **0.005** | **0.756** | **0.000** | **0.834** | **0.008** |
| Tb927.6.3800 | Heat shock 70 kDa protein | 71.4 | 5.8 | ATP-binding | **0.703** | **0.001** | 1.031 | 0.580 | **0.789** | **0.000** | **0.787** | **0.005** |
| Tb927.7.3330 | Hypothetical protein | 502.6 | 4.2 | ATP-binding, coiled coil, repeats | **0.703** | **0.000** | **0.765** | **0.000** | **0.889** | **0.006** | **0.803** | **0.000** |
| Tb11.02.5280 | Glycerol-3-phosphate dehydrogenase | 66.9 | 8.1 | DAO, FADG3PDH, FAD/NAD(P) binding, WXXXYIF motif | **0.666** | **0.001** | 1.037 | 0.553 | **0.614** | **0.000** | **0.633** | **0.000** |
| Tb09.160.1200 | GB4 mitotubule-associated protein | 928.3 | 4.3 | ConA-like lectin/gluconase, repeats, coiled coil | **0.557** | **0.016** | **0.553** | **0.006** | **0.738** | **0.013** | **0.689** | **0.003** |
| Tb09.211.3550 | glk1 glycerol kinase, glycosomal | 56.3 | 8.2 | FGGY, glycerol kinase, PTS2 pattern, actin-like ATPase domain | **0.540** | **0.000** | **0.865** | **0.000** | **0.672** | **0.000** | **0.656** | **0.000** |
| Tb10.70.5670 | TEF1 elongation factor 1-alpha | 49.1 | 9.4 | GTP-binding | **0.460** | **0.000** | **0.618** | **0.000** | **0.858** | **0.001** | **0.798** | **0.000** |
| Tb927.1.700 | PGKC phosphoglycerate kinase | 47.2 | 9.9 | PGK | **0.449** | **0.016** | **0.539** | **0.014** | **0.609** | **0.032** | 0.697 | 0.061 |
| Tb927.3.3270 | TbPFK ATP-dependent phosphofructokinase | 53.5 | 9.7 | PFK, ATP-binding, Rossmann fold, coiled coil, PTS1 pattern | 0.962 | 0.393 | **1.218** | **0.002** | **1.457** | **0.000** | **1.435** | **0.000** |
| Tb927.6.4670 | CMRP flagellar component | 40.6 | 5.1 | MORN repeats, PIP5K-related, PTB motif | 1.003 | 0.966 | **1.178** | **0.000** | 0.949 | 0.054 | 0.967 | 0.520 |
| Tb11.01.2800 | Hypothetical protein | 41.6 | 10.1 | Coiled coil | 1.067 | 0.400 | **1.164** | **0.005** | 0.995 | 0.918 | 0.977 | 0.764 |
| Tb11.01.3110 | Heat shock protein 70 | 75.3 | 6.3 | HSP70 peptide-binding domain, actin-like ATPase domain | 1.123 | 0.231 | **1.148** | **0.032** | 1.097 | 0.080 | **1.190** | **0.046** |
| Tb927.8.5010 | PFR2 69 kDa paraflagellar rod protein | 69.5 | 5.8 | Coiled coil | 1.027 | 0.543 | **1.142** | **0.000** | 1.011 | 0.722 | 0.953 | 0.135 |
| Tb927.3.4330 | PFR1 73 kDa paraflagellar rod protein; PFR1 | 68.6 | 5.9 | Coiled coil | 1.037 | 0.266 | **1.100** | **0.000** | **0.956** | **0.041** | **0.920** | **0.001** |
| Tb927.7.2650 | Hypothetical protein, conserved | 62.1 | 5.9 | Coiled coil, repeats | 0.860 | 0.090 | **0.899** | **0.044** | **1.319** | **0.001** | **1.185** | **0.031** |
| Tb11.01.8770 | Hypothetical protein, conserved | 110.1 | 4.3 | Leucine rich repeats | 0.937 | 0.477 | **0.828** | **0.006** | **1.261** | **0.002** | **1.199** | **0.004** |
| Tb927.6.5070 | Hypothetical protein, conserved | 51.8 | 6.1 | Coiled coil | 0.748 | 0.089 | **0.730** | **0.002** | 1.122 | 0.467 | 1.166 | 0.268 |
| Tb927.4.1300 | Hypothetical protein, conserved | 42.0 | 6.2 | Unknown | 1.028 | 0.656 | **0.722** | **0.000** | **2.133** | **0.000** | **2.015** | **0.000** |
| Tb11.02.0760 | Dynein heavy chain, putative | 531.1 | 5.2 | AAA+ ATPase core, coiled coil, thiol protease histidine active site | 0.710 | 0.180 | **0.668** | **0.044** | 0.784 | 0.224 | 0.793 | 0.176 |
| Tb11.02.0210 | Hypothetical protein, conserved | 50.8 | 4.9 | Coiled coil | 1.080 | 0.221 | 0.906 | 0.117 | **1.624** | **0.000** | **1.634** | **0.000** |
| Tb09.211.2150 | Poly(A)-binding protein 1; PABP2 | 62.1 | 10.1 | RNA recognition motif | 0.984 | 0.906 | 1.143 | 0.098 | **1.619** | **0.003** | **1.579** | **0.014** |
| Tb927.7.3550 | Hypothetical protein, conserved | 138.1 | 5.3 | C2 calcium-lipid binding motif | 1.131 | 0.152 | 0.990 | 0.889 | **1.349** | **0.000** | **1.391** | **0.000** |
| Tb927.8.8330 | Calpain, putative | 98.4 | 4.2 | Peptidase family C2. Proline-rich region | 1.067 | 0.224 | 1.020 | 0.666 | **1.339** | **0.000** | **1.392** | **0.000** |
| Tb09.160.1160 | NOP86 nucleolar protein | 85.9 | 4.4 | Coiled coil | 0.945 | 0.391 | 0.909 | 0.124 | **1.246** | **0.000** | **1.259** | **0.000** |
| Tb927.8.4580 | Hypothetical protein, conserved | 58.0 | 7.5 | Coiled coil. Nasopharyngeal epithelium specific protein 1 | 0.875 | 0.185 | 1.018 | 0.832 | **0.878** | **0.013** | 1.023 | 0.888 |
| Tb927.8.6660 | Hypothetical protein, conserved | 69.0 | 5.2 | Leucine-rich repeats, RNI-like | 0.965 | 0.502 | 1.051 | 0.303 | **0.833** | **0.000** | **0.837** | **0.000** |
