# Supplementary material for: The Orthologue of Sjögren's Syndrome Nuclear Autoantigen 1 (SSNA1) in Trypanosoma brucei Is an Immunogenic Self-Assembling Molecule
Source: PLoS One. 2012 Feb 20;7(2):e31842. doi: 10.1371/journal.pone.0031842 (PMC3282761; doi:10.1371/journal.pone.0031842)
Supplement: Methods S1 — Additional methods for comparative proteomic studies. (DOC) [file pone.0031842.s009.doc]

**ADDITIONAL MATERIALS AND METHODS**

***Subcellular Fractionation***

Cytoskeleton and flagellar fractions were prepared by subjecting cells to detergent/NaCl extraction as described previously [12]. Briefly, parasites were centrifuged at 800 *g* for 10 minutes at 20°C, then washed in PBS. Following centrifugation as above, cells were resuspended in PEME (100mM PIPES, 2mM EGTA, 0.1mM EDTA and 1mM MgSO_4_, pH 6.9) containing 1% Nonidet P40, 1 x Complete protease inhibitor cocktail (Roche), 7.5 μM Pepstatin A and 5μM E-64d. Parasites were incubated on ice for 10 minutes, then centrifuged at 15,000 *g* for 15 minutes at 4°C. Pellets were either washed twice in PEME and resuspended in Laemmli buffer (cytoskeleton fraction) or further extracted in PEME containing 1M NaCl, 200μg/ml DNaseI, 50μg/ml RNaseA and protease inhibitors as above. Samples were incubated on ice for 10 minutes, centrifuged as above, salt extraction repeated once, then pellets washed twice in PEME. Pellets were resuspended in Laemmli buffer (flagellar fraction). Total cell lysates, cytoskeletal and flagellar extracts from the equivalent of 1 x 10^7^ cells per sample were analysed by immunoblotting and probed with the following mouse monoclonal antibodies: anti-myc (Invitrogen, 1:2000), anti-elongation factor-1α (EF-1α) clone CBP-KK1 (Millipore, 1:2000) and anti-PFR1/2 clone L13D6 (a gift from Keith Gull, Sir William Dunn School of Pathology, University of Oxford, UK, 1:500).

***iTRAQ and Proteomic Data Analysis***

Flagellar extracts were prepared as detailed above from 5 x 10^8^ parasites of the following BSF lines: parental Lister 427 and cells expressing DIP13^GFP^ or DIP13^myc^ grown in the presence of tetracycline for 24 hours. DIP13^myc^ cell extracts were prepared in duplicate as experimental replicates. Extracted pellets were washed in PEME four times and redissolved in 200 μl of flagellum sample buffer (0.5M triethylammonium bicarbonate (TEAB), 5M urea, 0.5% SDS). Protein concentration was measured by Bradford assay (samples diluted 1:30 in PBS) and confirmed by densitometry of samples separated by SDS-PAGE and detected with SYPRO Ruby stain (Invitrogen). Protein samples (100 μg) were subjected to trypsin digestion and isobaric labelling using iTRAQ Reagents (Applied Biosystems), following the manufacturer’s instructions. The labelled samples were pooled, vacuum dried and reconstituted in 1ml of Strong Cation Exchange (SCX) Buffer A (10 mM potassium phosphate, 25% acetonitrile, pH2.7).

SCX was performed using a PolySULFOETHYL A 200 x 2.1 mm, 5 µm, 200 Å column (POLYLC inc) on an AKTA purifier 10 (GE). The flow rate used throughout was 0.1 ml/min. The column was pre equilibrated with a gradient of 0-100% buffer B (10 mM potassium phosphate, 25% acetonitrile, 1 M KCl, pH2.7) over 15 column volumes (CV), repeated three times. Following a further 5 CV of buffer A (as above), the labelled sample was loaded via a 2 ml sample loop, which was emptied with 4 ml buffer A. The column was washed with a further 2 CV of buffer A. Elution of bound peptides was achieved using a gradient of 0-15% Buffer B over 15 CV, 15-50% Buffer B over 7 CV, 50-80% Buffer B over 3 CV and 80-100% Buffer B over 5 CV. Fractions of 250 µl were collected in 96 well plates. Fractions were vacuum dried and reconstituted in 10 µl 0.1% TFA.

***Nano-LC-ESI-MS***

SCX fractions were loaded onto an Ultimate nano-LC system (Dionex) equipped with a PepMap C_18_ trap (300 µm x 0.5 cm, Dionex) and an Onyx C_18_ monolithic silica capillary column (100 μm x 15 cm, Phenomenex). The trap was equilibrated and washed with 0.1% (v/v) formic acid. The sample was loaded onto the trap at the start of the separation and then washed until the trap was switched in-line with the separating column. The separation used a gradient elution with the following conditions: the initial eluant composition was 100% Solvent A; on injection this was held for 3 min and then a 2-stage linear gradient of Solvent B from 0% to 20% in 40 min and 20% to 50% in 20 min was applied, followed by a 5 min wash at 95% Solvent B and finally re-equilibration in 100% Solvent A (Solvent A: 2% (v/v) acetonitrile, 0.1% (v/v) formic acid in H_2_O; Solvent B: acetonitrile, 0.1% (v/v) formic acid). The flow rate was 1.2 µL/min and the column temperature was 60°C. Control and data acquisition were with Chromeleon v6.6 software (Dionex). The nano-LC was interfaced with a QSTAR API Pulsar *i* Hybrid LC/MS/MS system (Applied Biosystems) with a MicroIonSpray source (fitted with 20 μm ID fused silica emitter, New Objective). Positive ESI MS & MS/MS spectra were acquired over the range 300 – 1800 m/z using information dependent acquisition (IDA). Analyst QS v1.1 software was used for instrument control, data acquisition and analysis. LC-MS/MS data files were analysed using ProteinPilot v2.0.1 software (Applied Biosystems). Peptides with an ion score with less than 95% confidence were excluded from the final dataset.
